# Supplementary material for: Targeted delivery of Fc-fused PD-L1 for effective management of acute and chronic colitis
Source: Nat Commun. 2024 Feb 23;15:1673. doi: 10.1038/s41467-024-46025-0 (PMC10891058; doi:10.1038/s41467-024-46025-0)
Supplement: Supplementary file 1 — Supplementary Information [file 41467_2024_46025_MOESM1_ESM.pdf]

Supplementary Information for

Targeted delivery of Fc-fused PD-L1 for effective management of acute and chronic colitis

Xudong Tang[#1], Yangyang Shang[#1,2], Hong Yang[#1],Yalan Song[1], Shan Li[1], Yusi Qin[1], Jingyi Song[1], Kang Chen[1], Yang Liu[3], Dinglin Zhang[*2], Lei Chen[*1]

[1] Institute of Gastroenterology of PLA, Southwest Hospital, Army Medical University (Third Military Medical University) Chongqing 400038, China

[2] Department of Chemistry, College of Basic Medicine, Army Medical University (Third Military Medical University), Chongqing 400038, China

[3] Department of Laboratory Animal Science, College of Basic Medicine, Army Medical University (Third Military Medical University), Chongqing 400038, China

[#] Equally Contributing Authors

[*] Corresponding authors

E-mail addresses: chenlei_1977603@126.com (Lei Chen), zh18108@tmmu.edu.cn (Dinglin Zhang)

Supplementary Figures

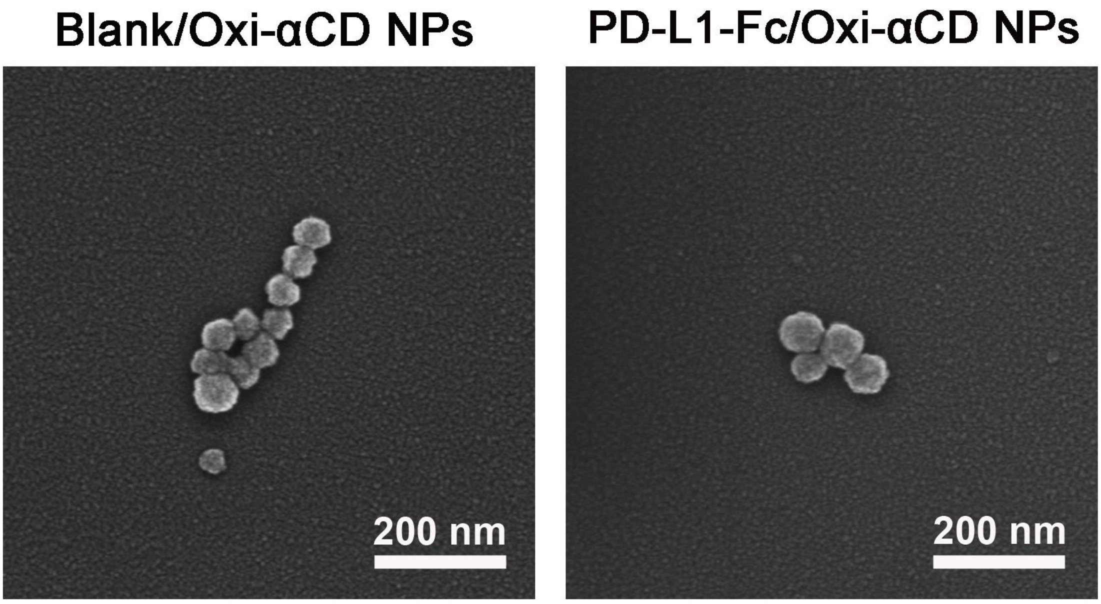

Supplementary Fig. 1 Scanning electron microscopy (SEM) images of Blank/Oxi-αCD nanoparticles and PD-L1-Fc/Oxi-αCD nanoparticles. $n = 3$ independent experiments. NPs nanoparticles.

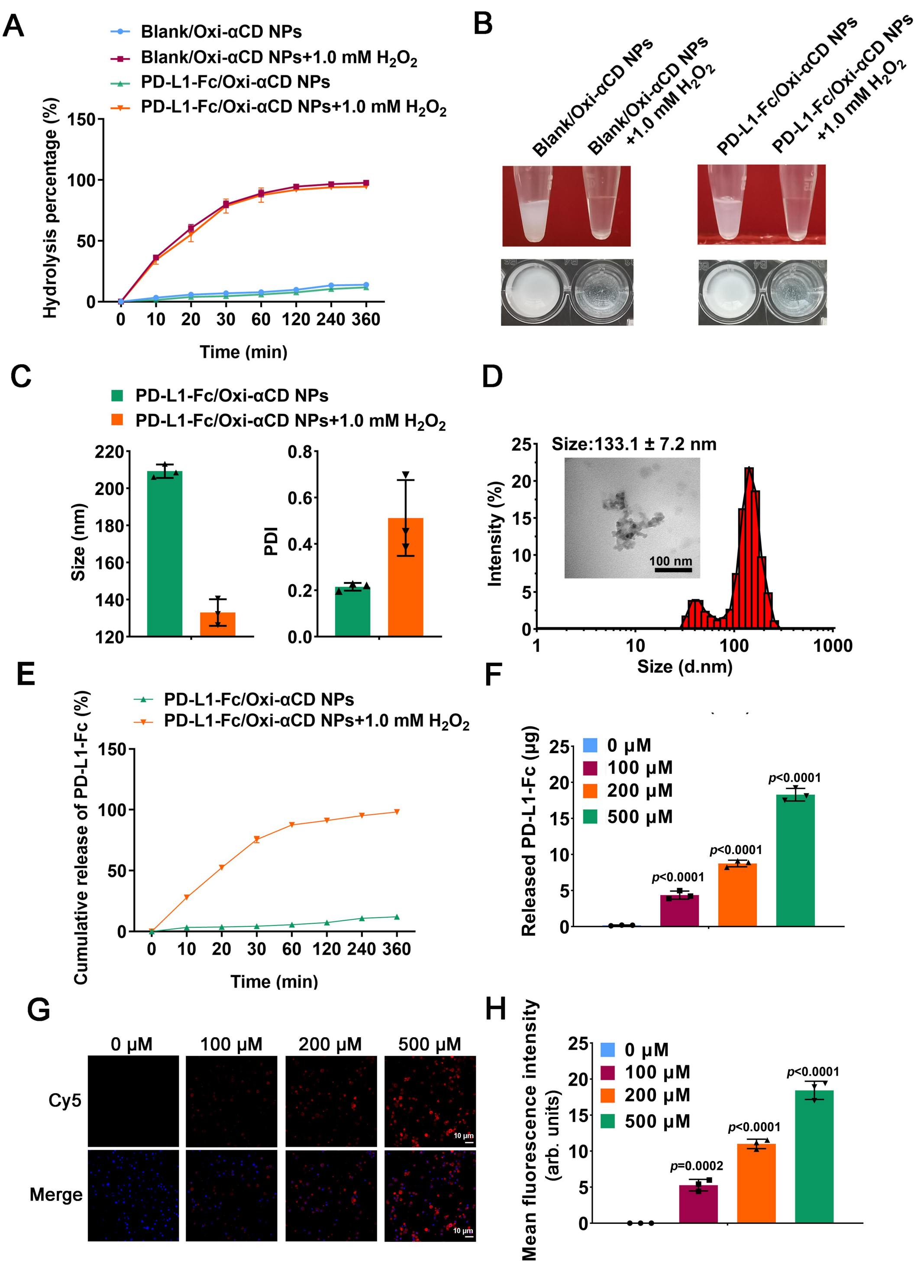

Supplementary Fig. 2 Hydrolysis and in vitro PD-L1-Fc release profiles of PD-L1-Fc/Oxi-αCD nanoparticles. **A**, **B** Hydrolysis curves (**A**) and images (**B**) of Blank/Oxi-αCD nanoparticles or PD-L1-Fc/Oxi-αCD nanoparticles with or without 1 mM H_2O_2 treatment. **C** Size (left) and PDI (right) of the PD-L1-Fc/Oxi-αCD nanoparticles with or without 1 mM H_2O_2 treatment. Data are presented as the mean ± SD. **D** Transmission electron microscopy (TEM) images and size distribution profile of PD-L1-Fc/Oxi-αCD nanoparticles treated with 1 mM H_2O_2 for 30 min. **E** In vitro release profiles of PD-L1-Fc with or without 1 mM H_2O_2 treatment. **F** PD-L1-Fc release within 1 h at different concentrations of H_2O_2 (n = 3 independent experiments, $p = 8.44227\times10^{-5}$, 3.41557×10^{-7} and 1.99307×10^{-9}). **G**, **H** CLSM

images (**G**) and quantitative data (**H**) of splenic lymphocytes treated with Cy5-PD-L1-Fc at different concentrations (n = 3 biologically independent samples, $p = 2.16412\times10^{-4}$, 8.77681×10^{-7} and 1.60518×10^{-8}). Blue channel, nucleus; Red channel, Cy5. Data are presented as mean ± SD (n = 3 independent experiments for **A**, **C**, **D**, **E**, **F**, and n = 3 biologically independent samples for **H**). P values derived from one-way ANOVA analysis followed by Tukey's multiple comparisons test (**F** and **H**). Source data are provided as a Source Data file. NPs nanoparticles.

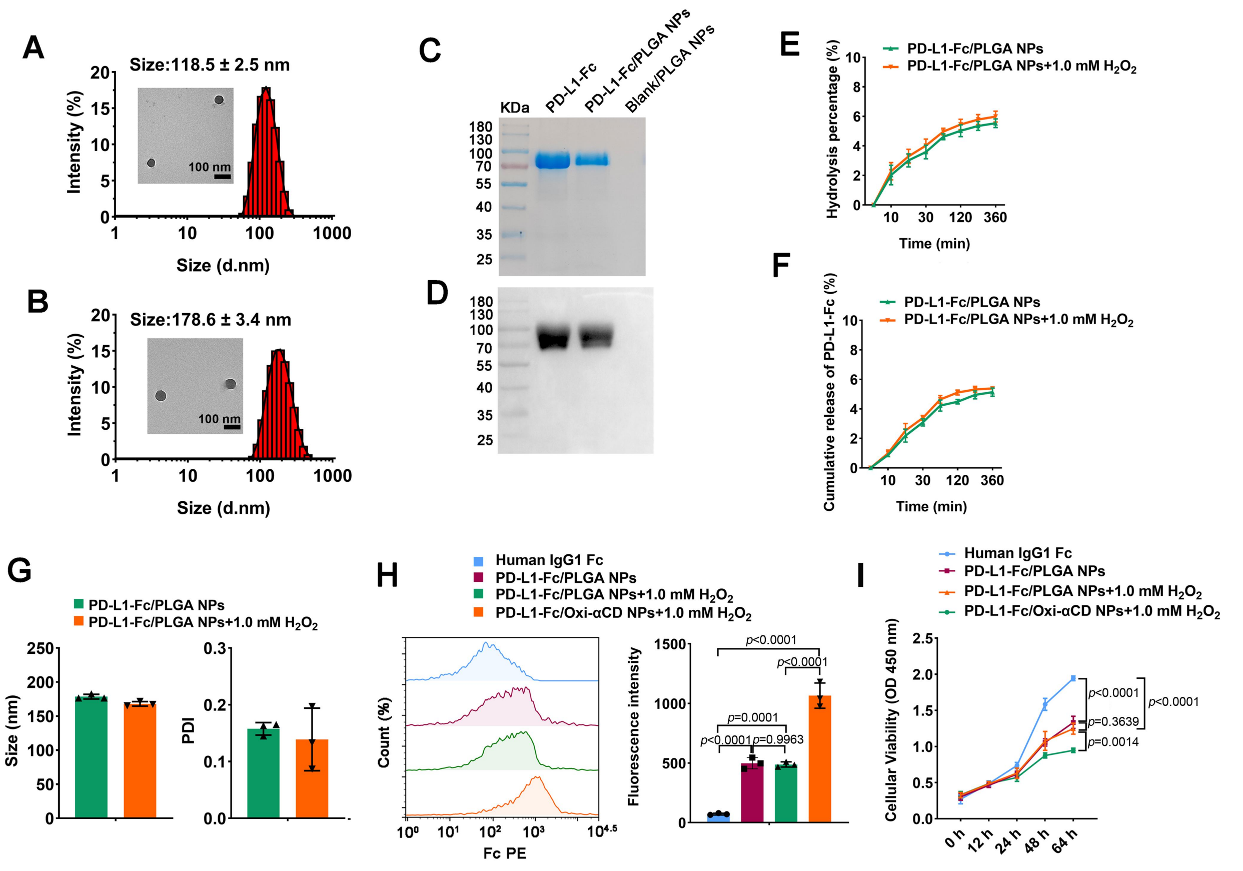

Supplementary Fig. 3 Characterization of the physicochemical properties of the PD-L1-Fc/PLGA nanoparticles. A, **B** Transmission electron microscopy (TEM) images and size distribution profile of PD-L1-Fc/PLGA nanoparticles. Scale bars = 100 nm. **C**, **D** Loading of the PD-L1-Fc protein on Blank/Oxi-αCD nanoparticles was confirmed by SDS–PAGE (**C**) and western blotting (**D**). **E**, **F** Hydrolysis profiles (**E**) and in vitro release profiles (**F**) of the PD-L1-Fc/PLGA nanoparticles with or without 1 mM H_2O_2 treatment. **G** Size (left) and PDI (right) of the PD-L1-Fc/PLGA nanoparticles with or without 1 mM H_2O_2 treatment. **H** Flow cytometry analysis of the binding capability of the PD-L1-Fc/Oxi-αCD nanoparticles with or without 1 mM H_2O_2 treatment to spleen lymphocytes (*n* = 3 biologically independent samples, $p = 9.87961\times10^{-5}$, 1.18045×10^{-4}, 1.24595×10^{-7}, 9.96259×10^{-1} and 1.03714×10^{-5}). . The fluorescence intensity of PE revealed the bioactivity of PD-L1-Fc after chemical conjugation to the nanoparticles in different treatments. Human IgG1 Fc was used as a negative control. **I** Cell Counting Kit-8 (CCK-8) analysis of the cellular viability of anti-CD3-activated spleen lymphocytes treated with hydrolyzed or unhydrolyzed PD-L1-Fc/PLGA nanoparticles (*n* = 3 biologically independent samples, $p = 8.52939\times10^{-6}$, 3.12964×10^{-6}, 3.63934×10^{-1} and 1.40177×10^{-3}). The experiments in (**A – G**) were repeated three times independently with similar results. Data are presented as mean ± SD (*n* = 3 independent experiments for **A**, **B**, **E**, **F**, **G**, and *n* = 3 biologically independent samples for **H**, **I**). *P* values derived from one-way ANOVA analysis followed by Tukey's multiple comparisons test (**H** and **I**). Source data are provided as a Source Data file. NPs nanoparticles.

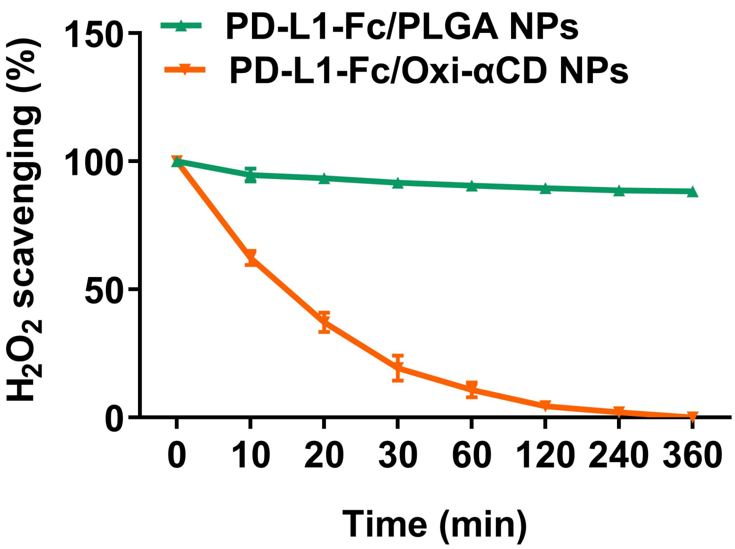

Supplementary Fig. 4 The ROS-scavenging curve of the nanoparticles. One milligram of PD-L1-Fc/Oxi-αCD nanoparticles or PD-L1-Fc/PLGA nanoparticles was treated with 1 mM H_2O_2 within 1 h, and residual H_2O_2 was monitored. Data are presented as the mean±SD (n = 3 independent experiments). Source data are provided as a Source Data file. NPs nanoparticles.

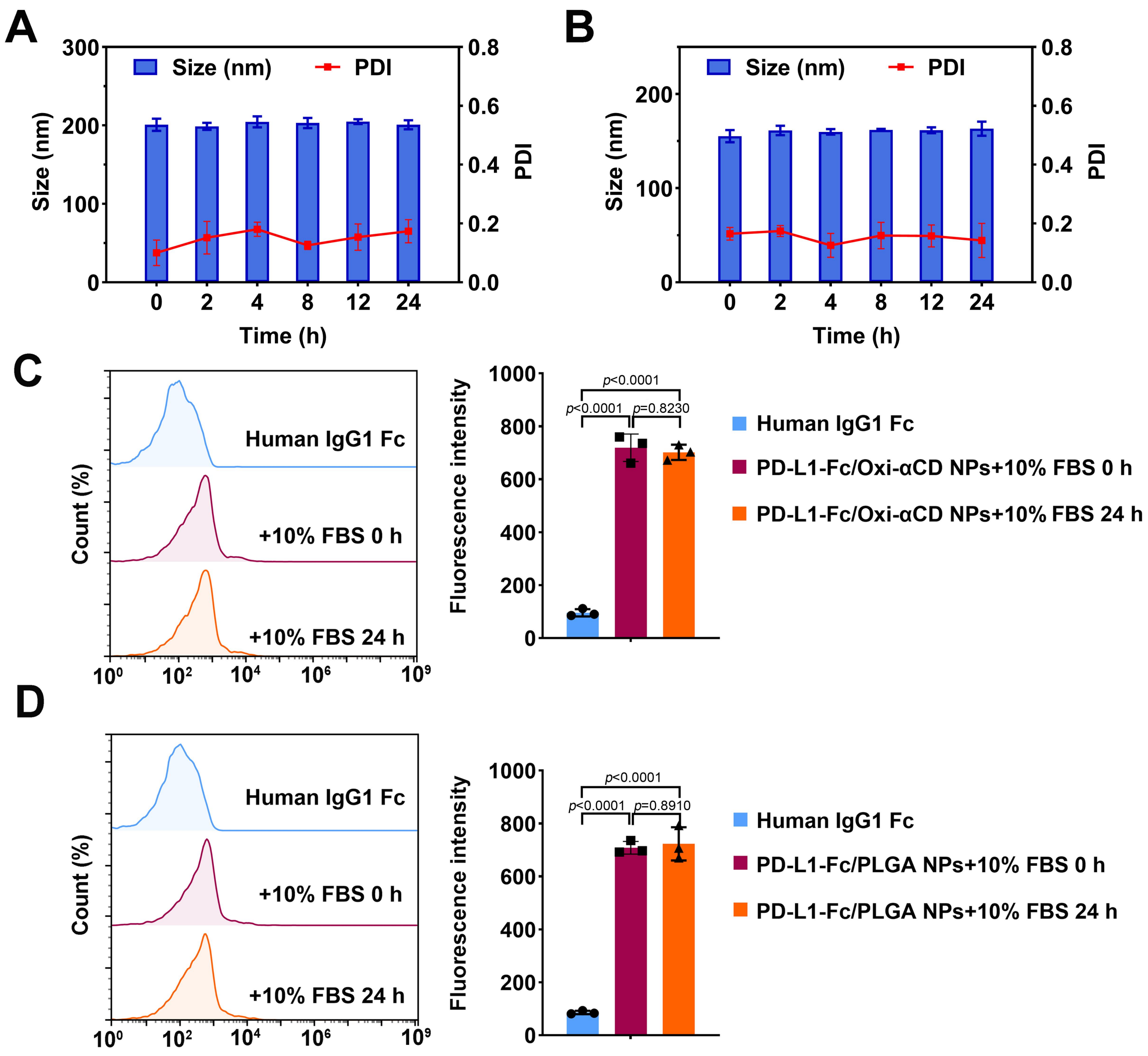

Supplementary Fig. 5 The stability of the nanoparticles and PD-L1-Fc in 10% FBS-containing medium. **A**, **B** DLS-determined size and PDI of PD-L1-Fc/Oxi-αCD nanoparticles (**A**) and PD-L1-Fc/PLGA nanoparticles (**B**). **C**, **D** Flow cytometry analysis of PD-L1-Fc binding to activated lymphocytes after nanoparticles incubation in 10% FBS-containing medium. In panel (**C**), $p = 1.53534\times10^{-6}$, 1.75300×10^{-6} and 8.23000×10^{-1}. In panel (**D**), $p = 2.58838\times10^{-6}$, 2.28108×10^{-6} and 8.91032×10^{-1}. The experiments in (**A**, **B**) were repeated three times independently with similar results. Data are presented as the mean±SD ($n = 3$ independent experiments for **A**, **B**, and $n = 3$ biologically independent samples for **C**, **D**). *P* values derived from one-way ANOVA analysis followed by Tukey's multiple comparisons test (**C** and **D**). Source data are provided as a Source Data file. NPs nanoparticles.

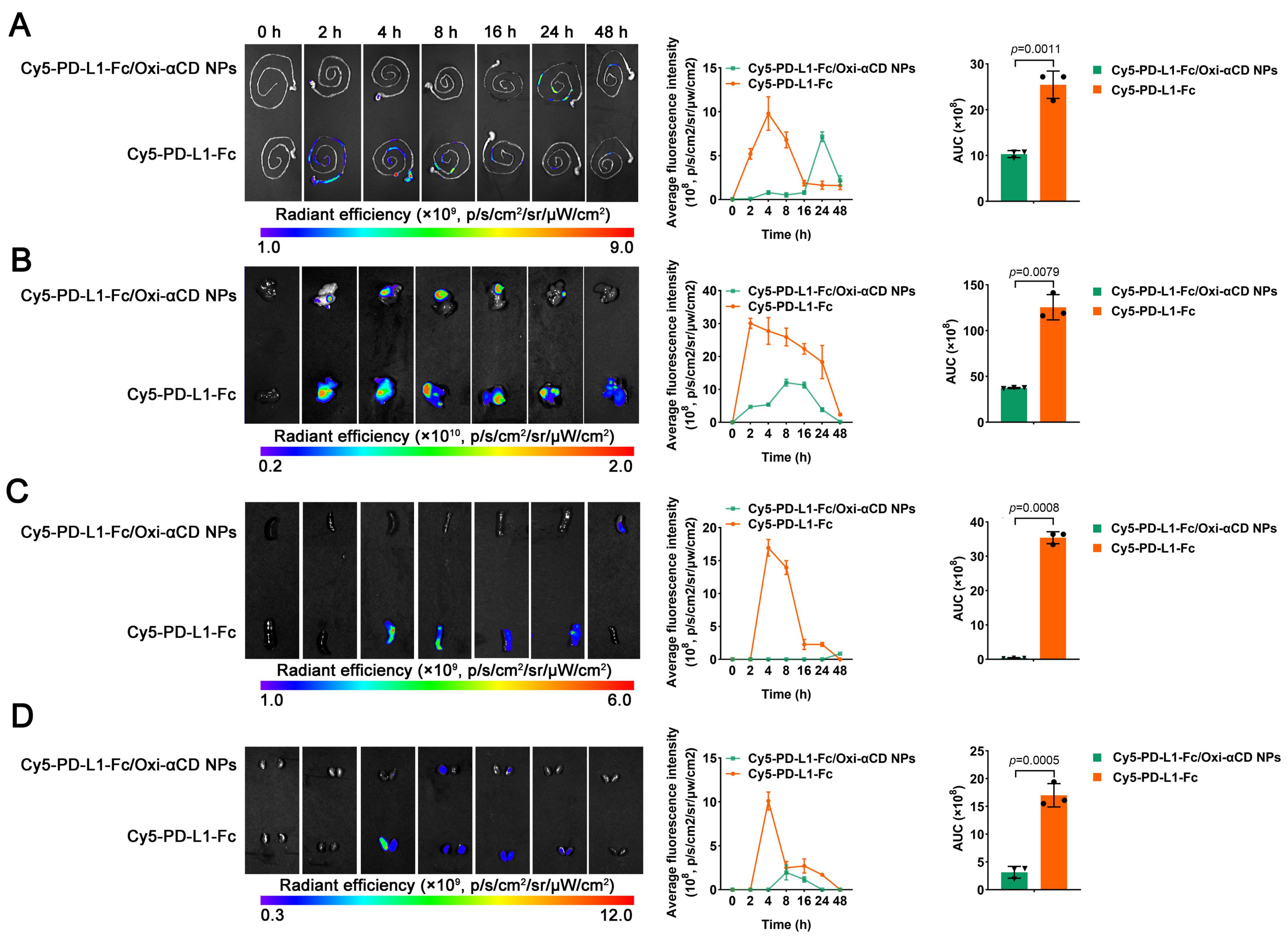

Supplementary Fig. 6 In vivo distribution of Cy5-labeled PD-L1-Fc and Cy5-labeled PD-L1-Fc/Oxi-αCD nanoparticles in mice with acute colitis. A–D Ex vivo images (left), quantitative data (middle), and the area under the fluorescence intensity-time curve (AUC) (right) of the intestine (**A**), liver (**B**), spleen (**C**), and kidney (**D**). In panel (**A**), $p = 1.05683\times10^{-3}$. In panel (**B**), $p = 7.91055\times10^{-3}$. In panel (**C**), $p = 7.94840\times10^{-4}$. In panel (**D**), $p = 5.22104\times10^{-4}$. Data are presented as the mean±SD ($n = 3$ mice). P values derived from two-sided Student's t-test (right panel of **A–D**). Source data are provided as a Source Data file. NPs nanoparticles.

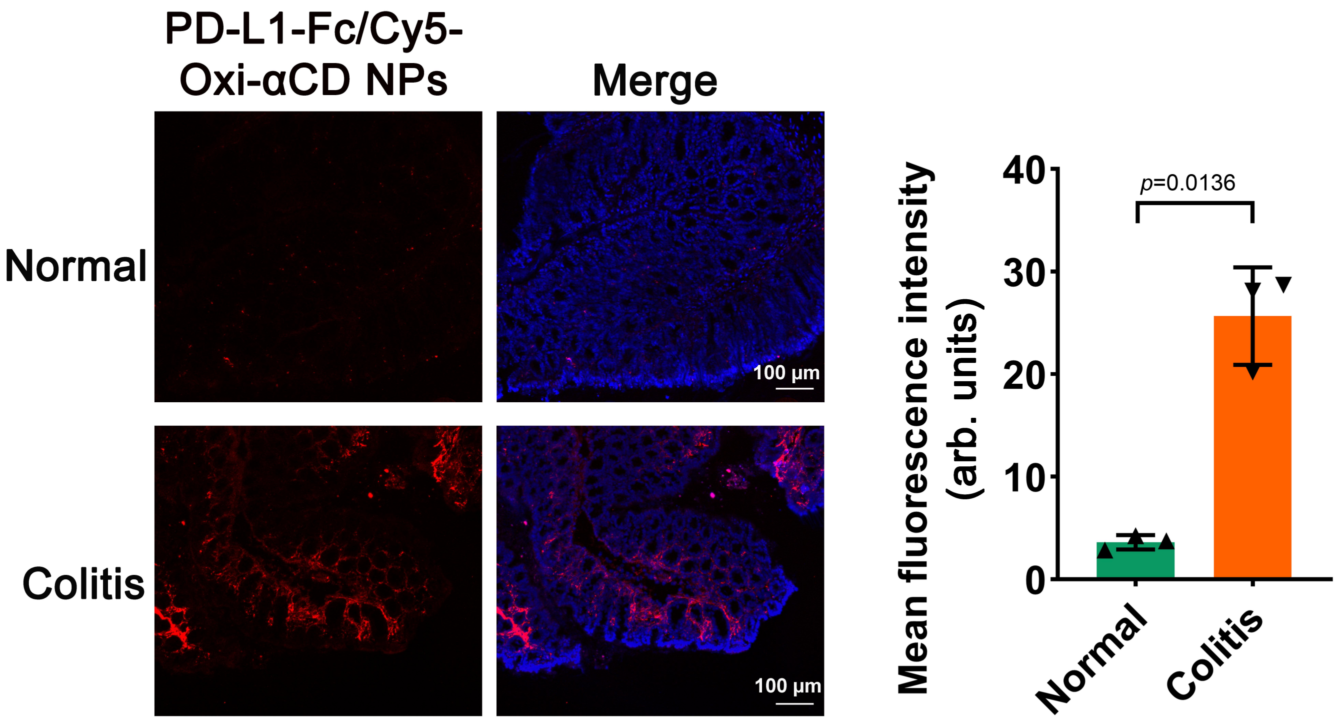

Supplementary Fig. 7 Fluorescence images of colonic cryosections from healthy mice and colitis mice at 8 hours after treatment with PD-L1-Fc/Cy5-Oxi-αCD nanoparticles. The red fluorescence indicates the presence of Cy5-labeled nanoparticles, while the blue fluorescence reveals DAPI-labeled nuclei. Data are presented as the mean±SD (n = 3 mice, $p = 1.35643\times10^{-2}$). P values derived from two-sided Student's t-test. Source data are provided as a Source Data file. NPs nanoparticles.

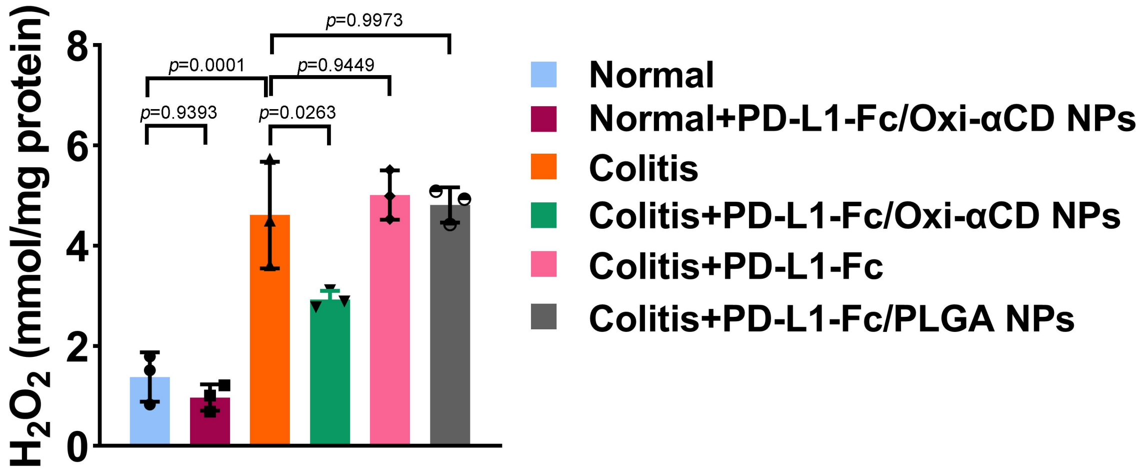

Supplementary Fig. 8 H_2O_2 level in colonic tissues from mice treated with different formulations. The level of H_2O_2 in colonic tissues isolated from healthy or colitis mice treated with PD-L1-Fc/Oxi-αCD nanoparticles, PD-L1-Fc or PD-L1-Fc/ PLGA nanoparticles for 8 h (n = 3 mice, p = 9.39269×10^{-1}, 1.29763×10^{-4}, 2.63386×10^{-2}, 9.44929×10^{-1} and 9.97300×10^{-1}). Data are presented as the mean±SD. P values derived from one-way ANOVA analysis followed by Tukey's multiple comparisons test. Source data are provided as a Source Data file. NPs nanoparticles.

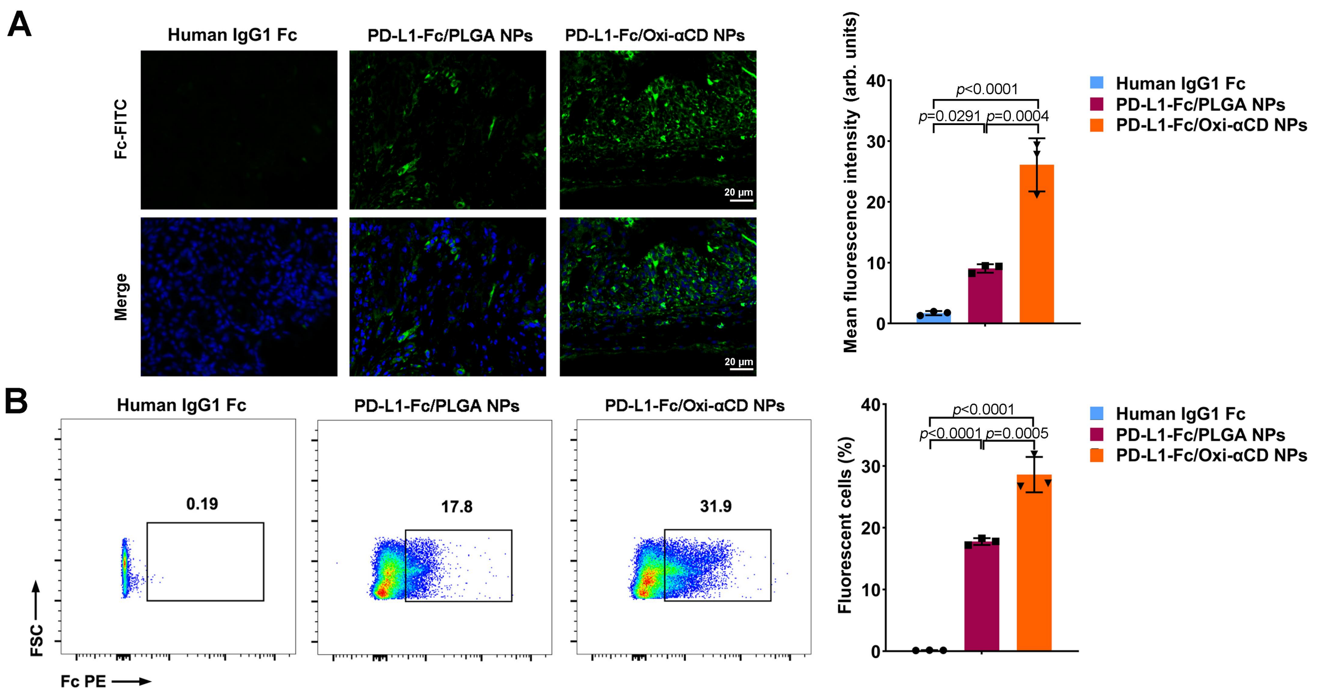

Supplementary Fig. 9 The distribution of PD-L1-Fc delivered by nanoparticles in the colon of colitis mice. A CLSM images (left) and quantitative data (right) of frozen colonic sections from mice treated with different formulations for approximately 8 h ($n = 3$ mice, $p = 2.90980\times10^{-2}$, 5.87526×10^{-5} and 4.47742×10^{-4}). Blue channel, nucleus; green channel, PD-L1-Fc. **B** Flow cytometry analysis of the distribution of PD-L1-Fc delivered by different formulations in colonic lamina propria cells from normal or colitis mice ($n = 3$ mice, $p = 3.45835\times10^{-5}$, 1.96937×10^{-6} and 5.47060×10^{-4}). Data are presented as the mean±SD. *P* values derived from one-way ANOVA analysis followed by Tukey's multiple comparisons test. Source data are provided as a Source Data file. NPs nanoparticles.

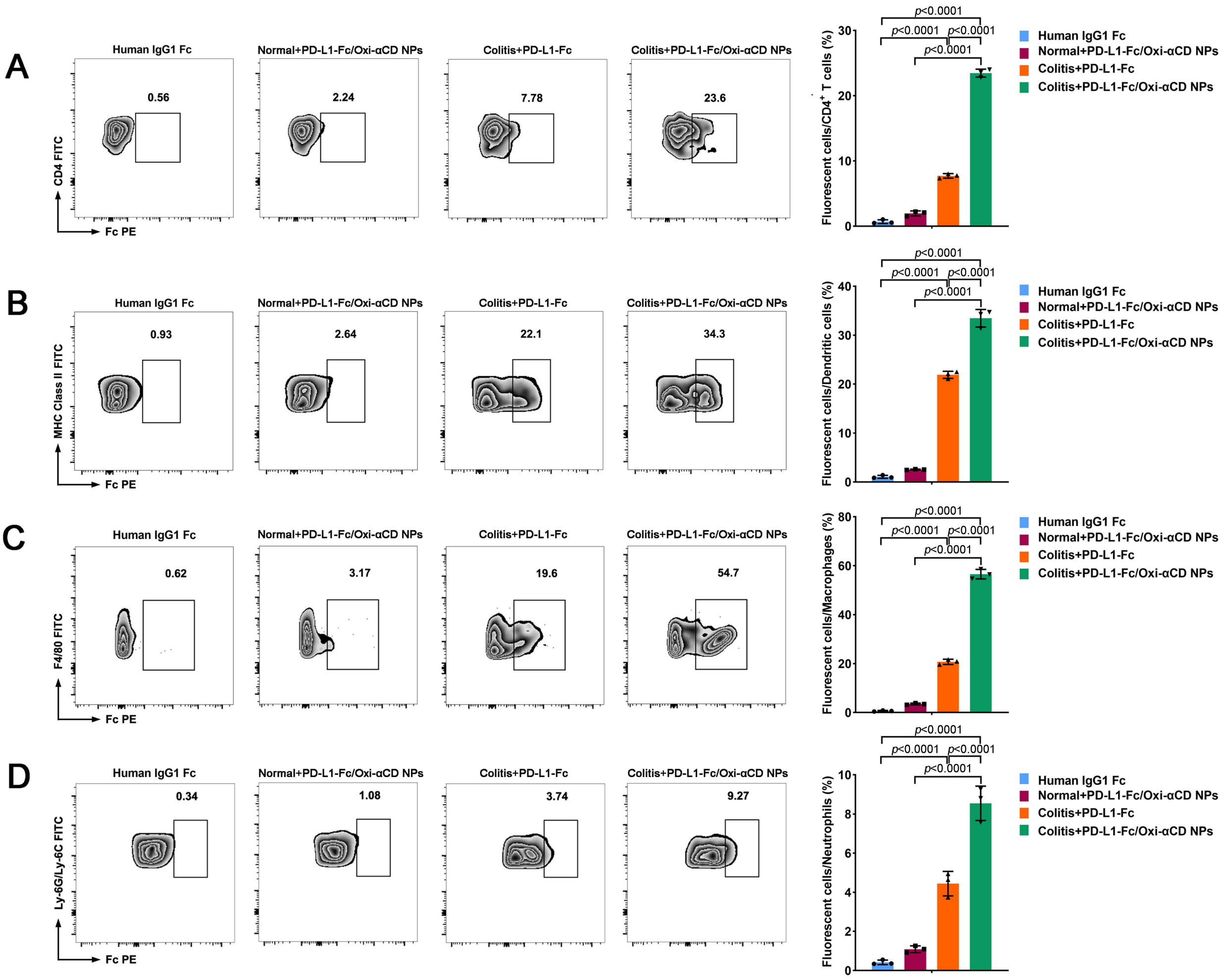

Supplementary Fig. 10 Distribution of PD-L1-Fc in immune cells of the colonic lamina propria from normal or colitis mice. A – D After 8 hours of treatment with PD-L1-Fc or PD-L1-Fc/Oxi-αCD nanoparticles, their distribution in CD4$^+$ T cells (**A**), dendritic cells (**B**), macrophages (**C**) and neutrophils (**D**) was measured by flow cytometry. In panel (**A**), p =1.41722 × 10^{-7}, 9.04000 × 10^{-13}, 3.14100 × 10^{-12} and 4.35220×10^{-10}. In panel (**B**), p =2.19017×10^{-8}, 1.46243×10^{-9}, 2.18367×10^{-9} and 2.52659×10^{-6}. In panel (**C**), p =7.45089 × 10^{-8}, 5.18400 × 10^{-12}, 1.41280 × 10^{-11} and 2.05454 × 10^{-9}. In panel (**D**), p =8.69808 × 10^{-5}, 3.91743×10^{-7}, 8.16927×10^{-7} and 7.48708×10^{-5}. Data are presented as the mean±SD (n = 3 mice). P values derived from one-way ANOVA analysis followed by Tukey's multiple comparisons test. Source data are provided as a Source Data file. NPs nanoparticles.

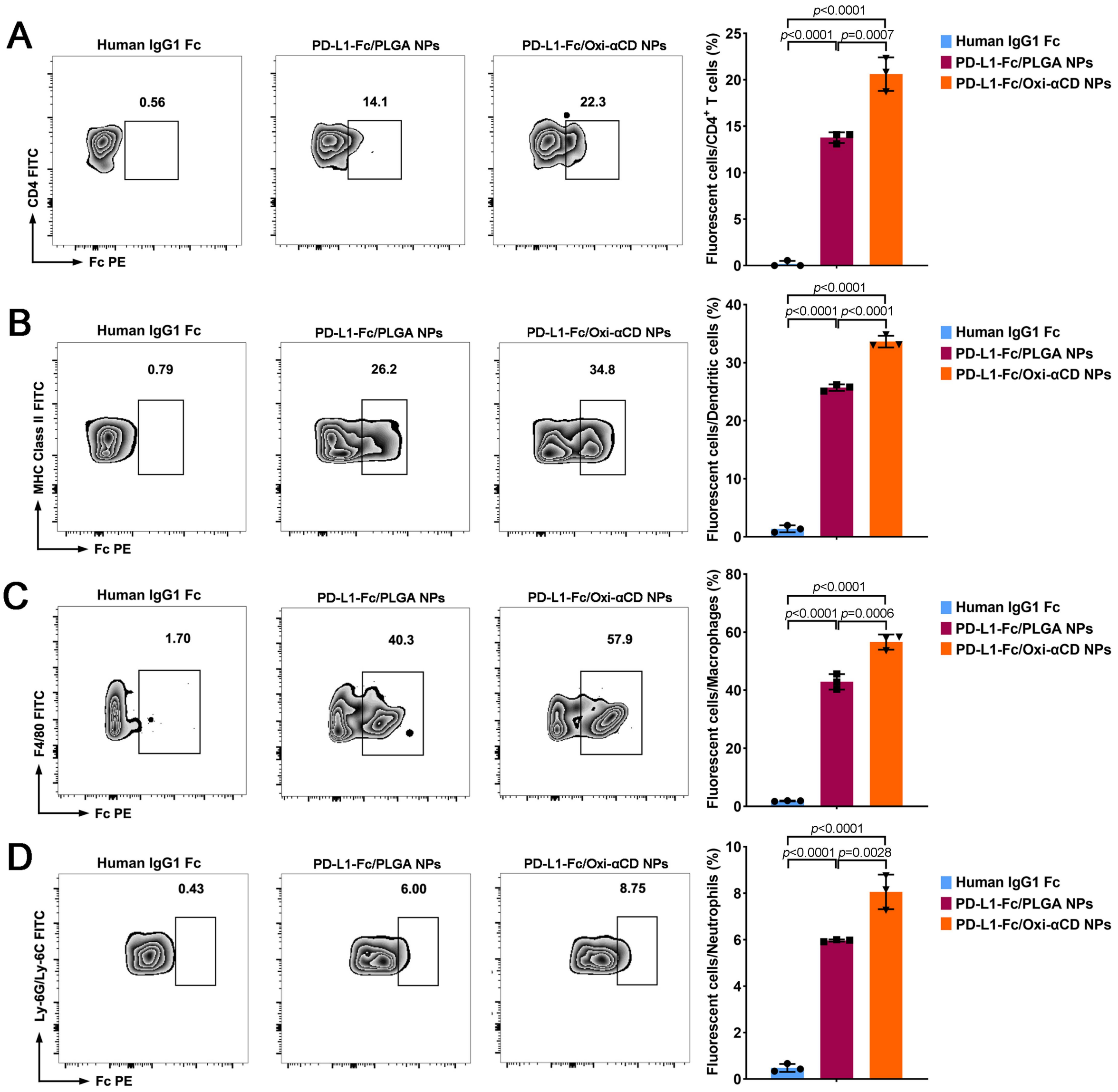

Supplementary Fig. 11 Distribution of PD-L1-Fc/PLGA nanoparticles or PD-L1-Fc/Oxi-αCD nanoparticles in immune cells in the colonic lamina propria of colitis mice. A – D After 24 hours of treatment with PD-L1-Fc or PD-L1-Fc/Oxi-αCD nanoparticles, their distribution in $CD4^+$ T cells (**A**), dendritic cells (**B**), macrophages (**C**) and neutrophils (**D**) was measured by flow cytometry. In panel (**A**), p =1.35067×10^{-5}, 1.32337×10^{-6} and 6.96334×10^{-4}. In panel (**B**), p =5.42493×10^{-8}, 1.87432×10^{-9} and 3.20389×10^{-5}. In panel (**C**), p =1.11763×10^{-6}, 3.02708×10^{-7} and 5.68329×10^{-4}. In panel (**D**), p =1.22395×10^{-5}, 1.81089×10^{-6} and 2.77616×10^{-3}. Data are presented as the mean±SD (n = 3 mice). P values derived from one-way ANOVA analysis followed by Tukey's multiple comparisons test. Source data are provided as a Source Data file. NPs nanoparticles.

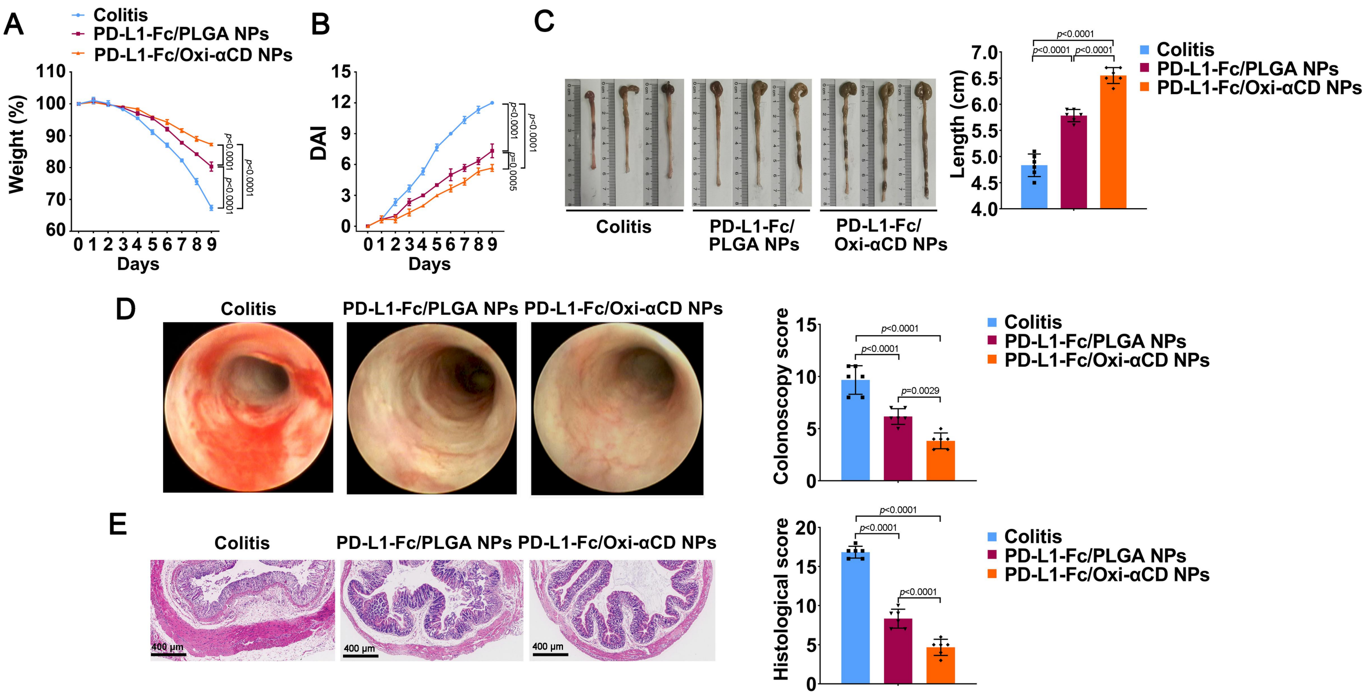

Supplementary Fig. 12 Therapeutic effect of PD-L1-Fc/PLGA nanoparticles versus PD-L1-Fc/Oxi-αCD nanoparticles on acute DSS-induced colitis. A Changes in body weight of mice in each group during 9 days of treatment (n = 6 mice, p = 1.00000×10^{-15}, 1.00000×10^{-15} and 5.59430×10^{-11}). Data were normalized to the percentage of body weight on day 0. **B** DAI of mice in each group during treatment (n = 6 mice, p = 3.98549×10^{-10}, 8.00000×10^{-12} and 5.01761×10^{-4}). **C** Representative digital photos (left panel) and quantified lengths (right panel) of colonic tissues isolated from mice after treatment (n = 6 mice, p = 1.69285×10^{-7}, 4.57190×10^{-11} and 2.57644×10^{-6}). **D** Representative mini-endoscopic images of colons from mice in different treatment groups. The right panel indicates the quantification of the severity of DSS-induced acute colitis (n = 6 mice, p = 6.10696×10^{-5}, 1.24957×10^{-7} and 2.87768×10^{-3}). **E** H&E-stained histological sections of colons. The right panel indicates the pathology score of colons from mice in the different treatment groups (n = 6 mice, p = 9.09713×10^{-10}, 5.76100×10^{-12} and 4.39442×10^{-5}). Data are presented as mean ± SD. P values derived from One-way ANOVA analysis followed by Tukey's multiple comparisons test. Source data are provided as a Source Data file. NPs nanoparticles.

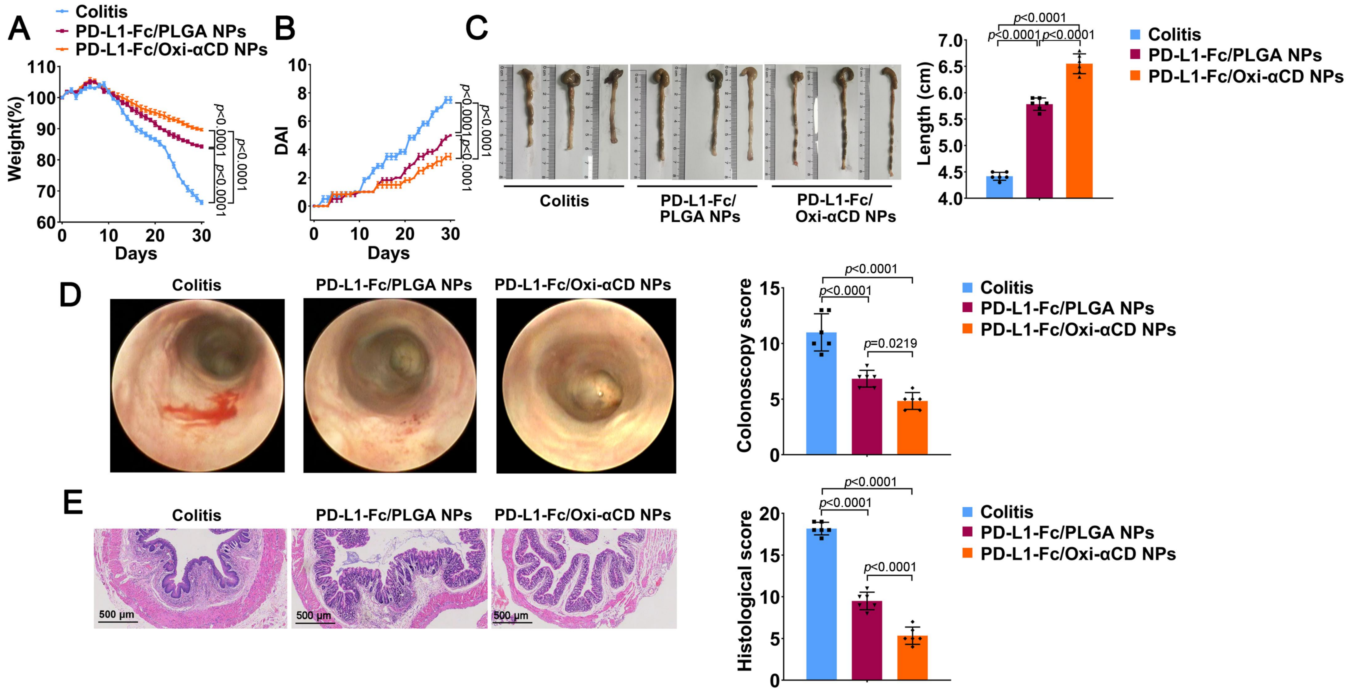

Supplementary Fig. 13 Effects of PD-L1-Fc/PLGA nanoparticles versus PD-L1-Fc/Oxi-αCD nanoparticles on the development of DSS-induced chronic colitis. **A** Changes in the body weight of mice in each group during 32 days of treatment (n = 6 mice, p = 1.00000×10^{-15}, 1.00000×10^{-15} and 1.97910×10^{-11}). Data were normalized to the percentage of body weight on day 0. **B** DAI of mice in each group during treatment (n = 6 mice, p = 2.18263×10^{-7}, 3.44177×10^{-10} and 9.63399×10^{-5}). **C** Representative digital photos (left panel) and quantified lengths (right panel) of colonic tissues isolated from mice after treatment (n = 6 mice, p = 5.56380×10^{-11}, 1.00000×10^{-15} and 1.70519×10^{-7}). **D** Representative mini-endoscopic images of colons from mice in the different treatment groups. The right panel indicates the quantification of the severity of DSS-induced acute colitis (n = 6 mice, p = 3.98805×10^{-5}, 3.53924×10^{-7} and 2.18743×10^{-2}). **E** H&E-stained histological sections of colons. The right panel indicates the pathology score of colons from mice in the different treatment groups (n = 6 mice, p = 2.76690×10^{-10}, 5.87000×10^{-13} and 4.86767×10^{-6}). Data are presented as mean ± SD. P values derived from One-way ANOVA analysis followed by Tukey's multiple comparisons test. Source data are provided as a Source Data file. NPs nanoparticles.

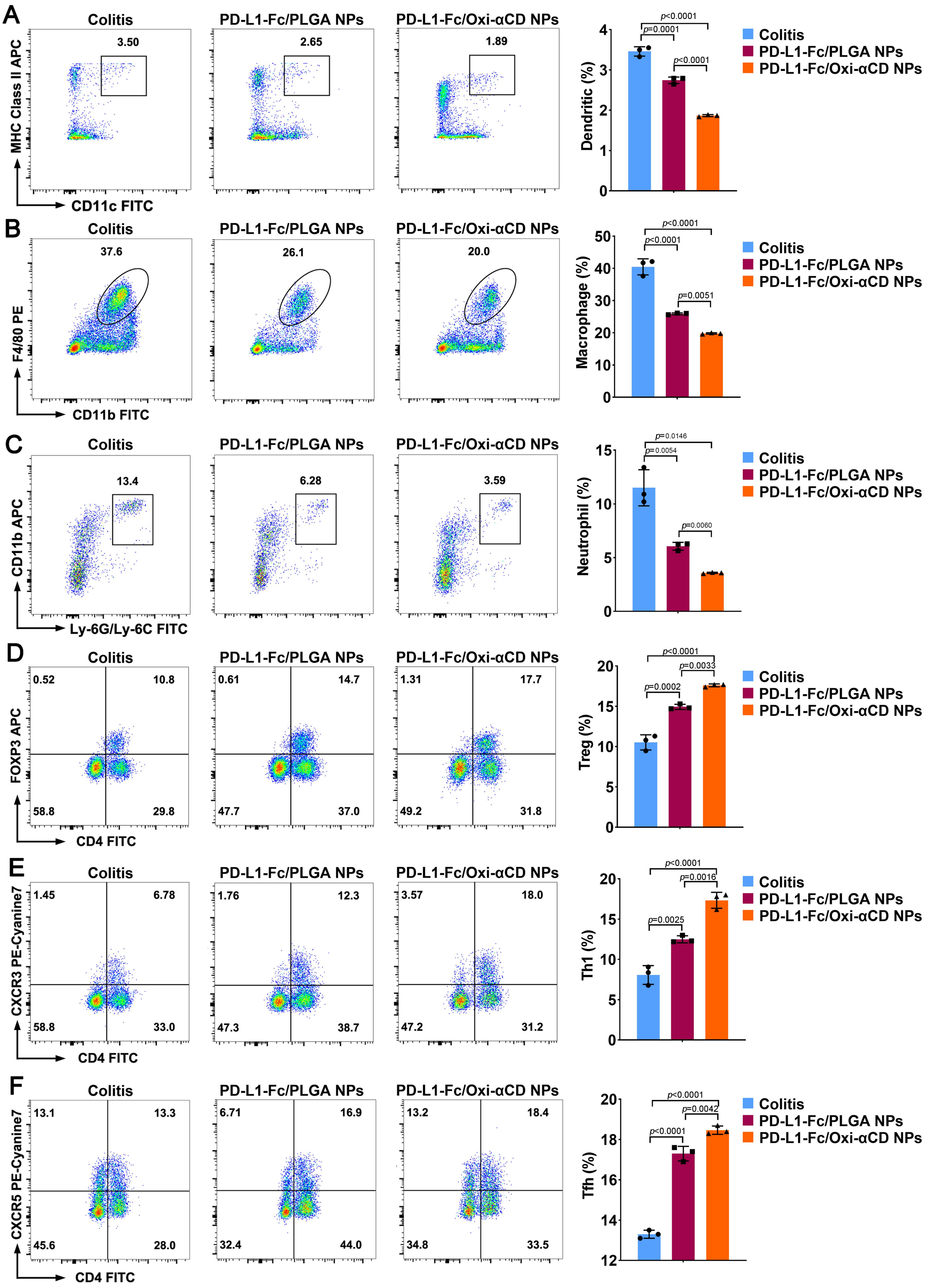

Supplementary Fig. 14 The regulatory effect of PD-L1-Fc/PLGA nanoparticles versus PD-L1-Fc/Oxi-αCD nanoparticles on the frequency of inflammatory cells derived from colonic tissues of mice with acute colitis. A – F Five days post-DSS administration, the frequencies of DCs(**A**), macrophages (**B**), neutrophils (**C**), Treg (**D**), Th1 (**E**), and Tfh (**F**) cells in isolated colonic lamina propria

(LP) cells from the indicated groups were determined by flow cytometry. In panel (**A**), $p = 1.00904\times10^{-4}$, 1.07498×10^{-6} and 3.14002×10^{-5}. In panel (**B**), $p = 4.34442\times10^{-5}$, 5.18402×10^{-6} and 5.07946×10^{-3}. In panel (**C**), $p = 5.40008\times10^{-3}$, 1.46293×10^{-2} and 6.04639×10^{-3}. In panel (**D**), $p = 2.19515\times10^{-4}$, 1.39866×10^{-5} and 3.30882×10^{-3}. In panel (**E**), $p = 2.53281\times10^{-3}$, 4.23284×10^{-5} and 1.60526×10^{-3}. In panel (**F**), $p = 3.78172\times10^{-6}$, 1.05584×10^{-6} and 4.16731×10^{-3}. Data are presented as mean ± SD ($n = 3$ mice). *P* values derived from One-way ANOVA analysis followed by Tukey's multiple comparisons test. Source data are provided as a Source Data file. NPs nanoparticles.

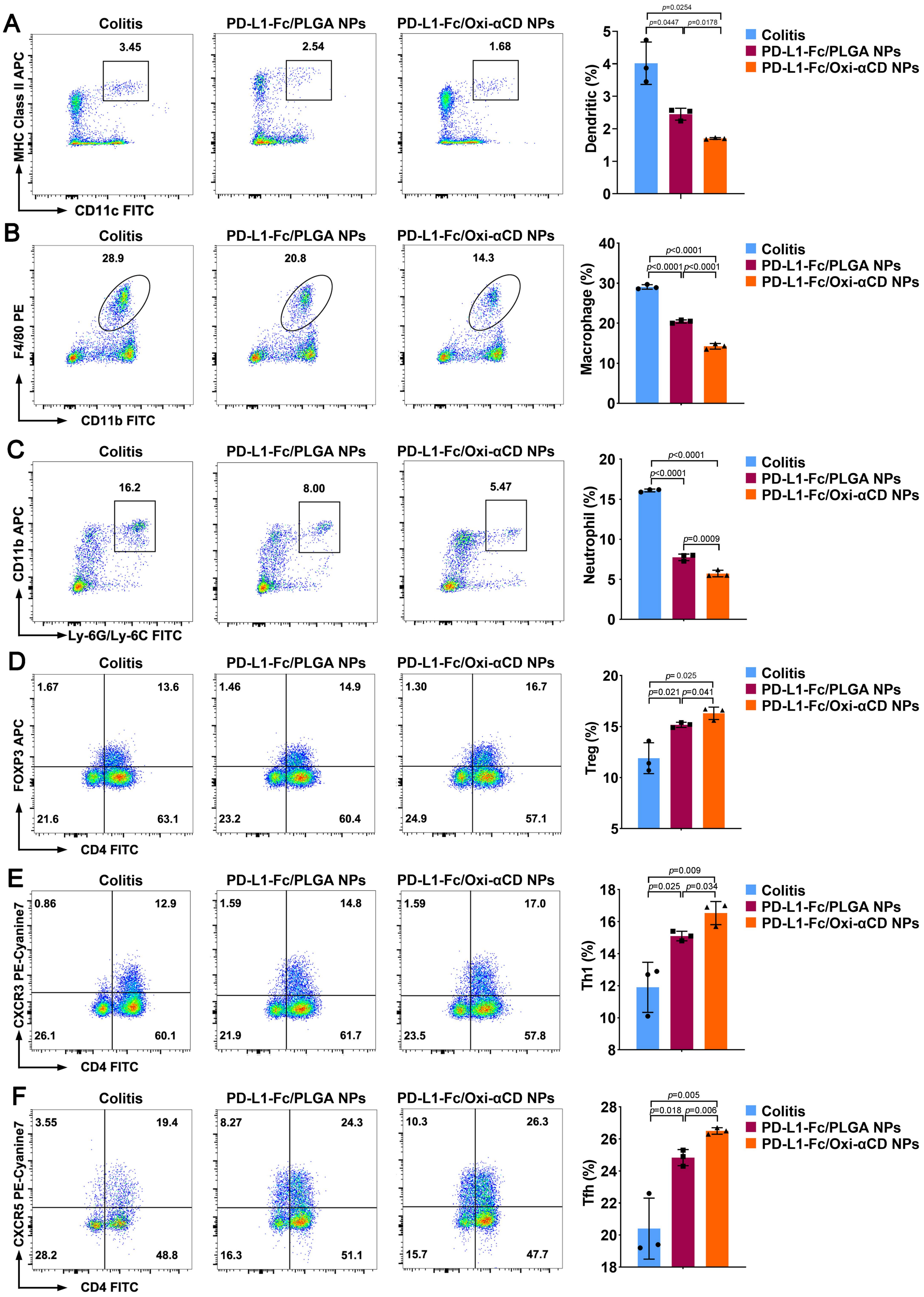

Supplementary Fig. 15 The regulatory effect of PD-L1-Fc/PLGA nanoparticles versus PD-L1-Fc/Oxi-αCD nanoparticles on the frequency of inflammatory cells derived from colonic tissues of mice with chronic colitis. A−F After three cycles of DSS administration, the frequencies of DCs (**A**),

macrophages (**B**), neutrophils (**C**), Treg (**D**), Th1 (**E**), and Tfh (**F**) cells in isolated colonic lamina propria (LP) cells from the indicated groups were determined by flow cytometry. In panel (**A**), $p = 4.46585\times10^{-2}$, 2.53622×10^{-2} and 1.78159×10^{-2}. In panel (**B**), $p = 3.62681\times10^{-6}$, 2.73113×10^{-7} and 2.76084×10^{-5}. In panel (**C**), $p = 4.17911\times10^{-7}$, 1.09375×10^{-7} and 9.49116×10^{-4}. In panel (**D**), $p = 2.10505\times10^{-2}$, 2.50268×10^{-2} and 4.06585×10^{-2}. In panel (**E**), $p = 2.52468\times10^{-2}$, 9.57590×10^{-3} and 3.38577×10^{-2}. In panel (**F**), $p = 1.76664\times10^{-2}$, 5.30146×10^{-3} and 5.96519×10^{-3}. Data are presented as mean ± SD ($n = 3$ mice). *P* values derived from One-way ANOVA analysis followed by Tukey's multiple comparisons test. Source data are provided as a Source Data file. NPs nanoparticles.

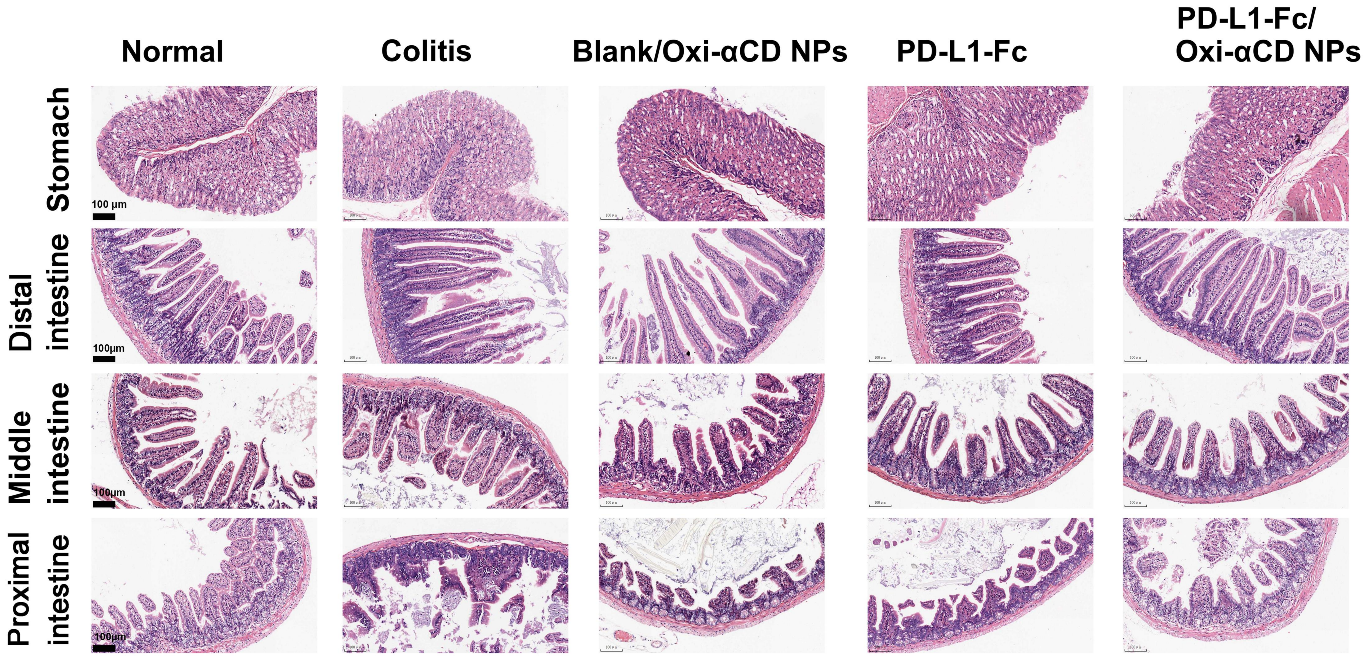

Supplementary Fig. 16 H&E-stained histological sections of gastrointestinal tissues from mice with chronic enteritis treated with different formulations. Histological analyses of H&E-stained sections of the stomach, proximal intestine, middle intestine and distal intestine showed no detectable injuries in the PD-L1-Fc/Oxi-αCD nanoparticles-treated groups. NPs nanoparticles.

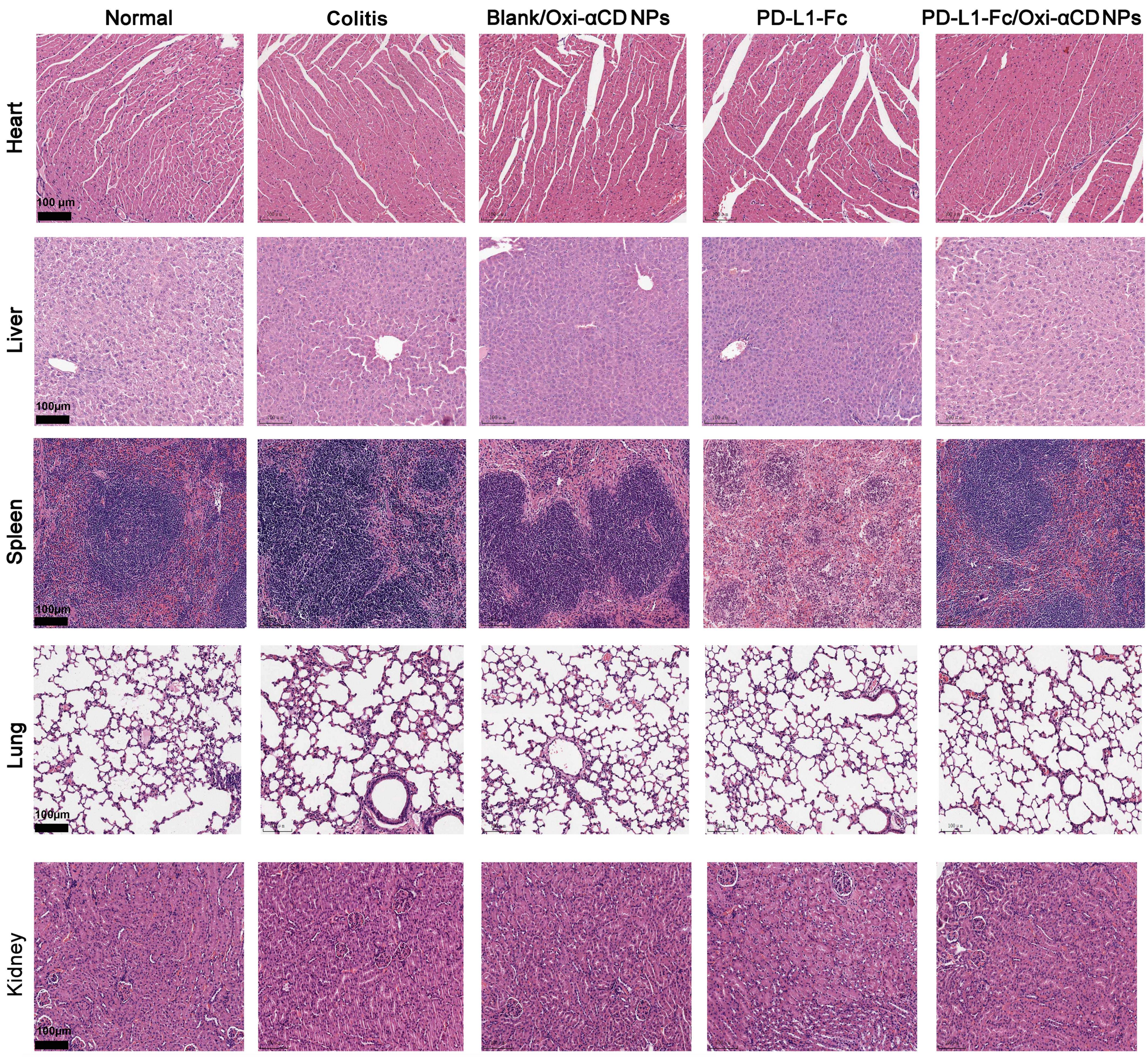

Supplementary Fig. 17 H&E-stained histological sections of representative major organs from mice with chronic enteritis treated with different formulations. H&E-stained sections of heart, liver, spleen, lung and kidney showed no detectable injuries in the PD-L1-Fc/Oxi-αCD nanoparticles-treated group, whereas spleens from chronic colitis mice treated with PD-L1-Fc showed a decrease in white pulp. NPs nanoparticles.

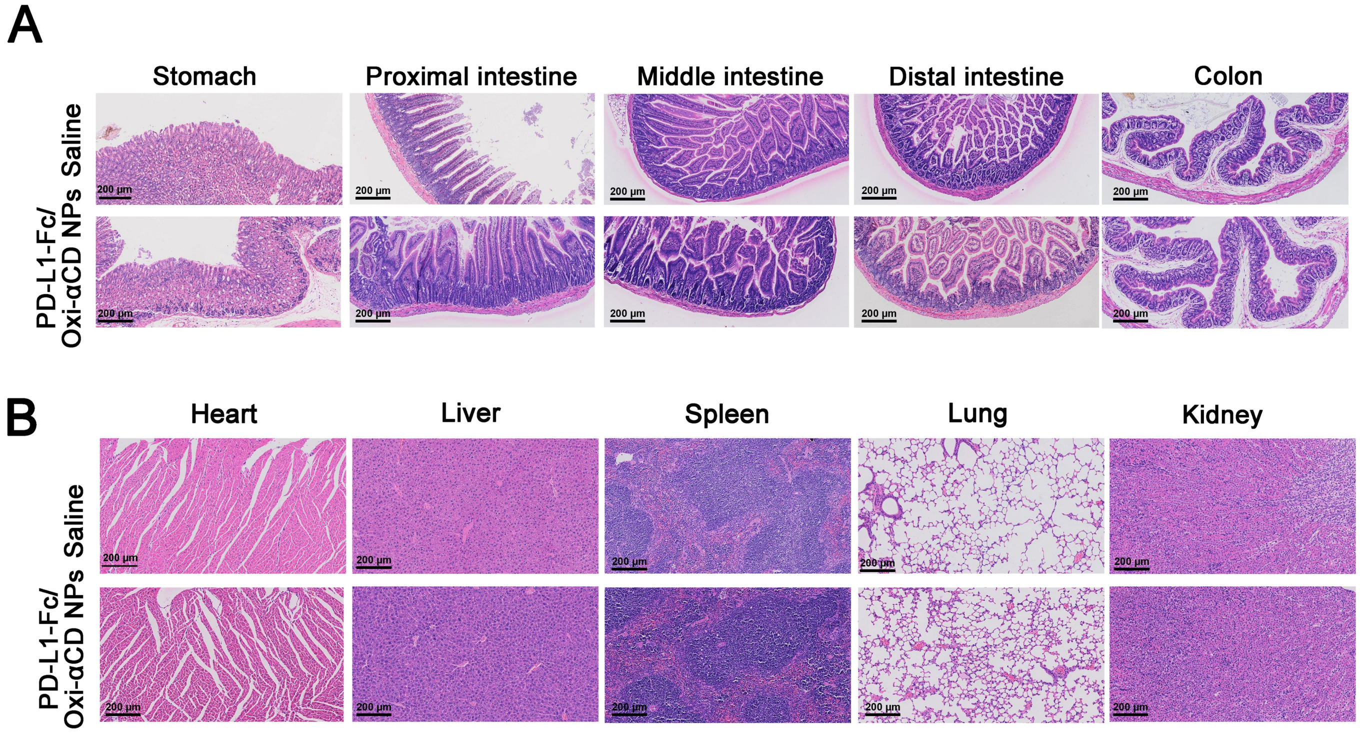

Supplementary Fig. 18 H&E-stained histological sections of the major organs from mice treated with a high dose of PD-L1-Fc/Oxi-αCD nanoparticles. A, **B** H&E-stained sections of gastrointestinal tissues (**A**) and major organs (**B**). Gastrointestinal tissues and major organs were resected from mice on day 12 after intraperitoneal injection of 5 g/kg PD-L1-Fc/Oxi-αCD nanoparticles. NPs nanoparticles.

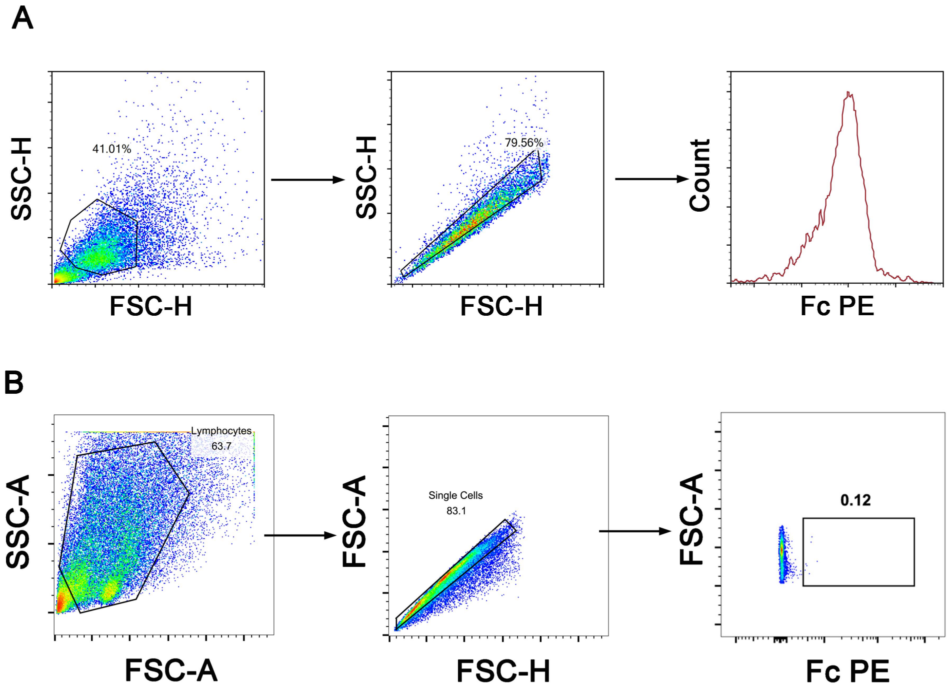

Supplementary Fig. 19 Gating strategy for the FACS tests. Panel A was applied for Fig. 2E, Supplementary Fig. 3H, Supplementary Fig. 5C and Supplementary Fig. 5D. Panel B was applied for Fig. 2K and Supplementary Fig. 9B.

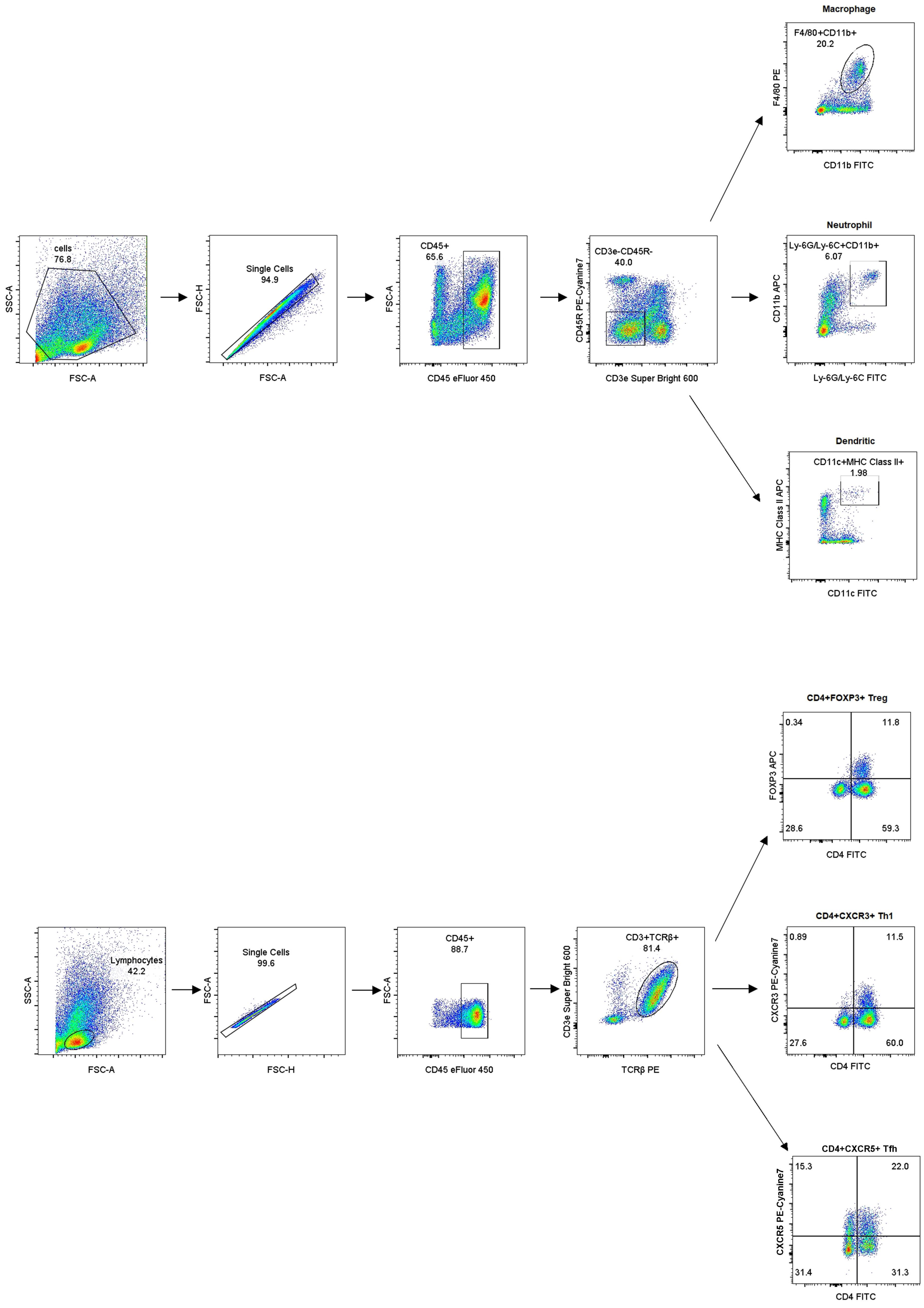

Supplementary Fig. 20 Gating strategy for the FACS tests applied for Fig. 5, Fig. 6, Supplementary Fig. 14 and Supplementary Fig. 15.

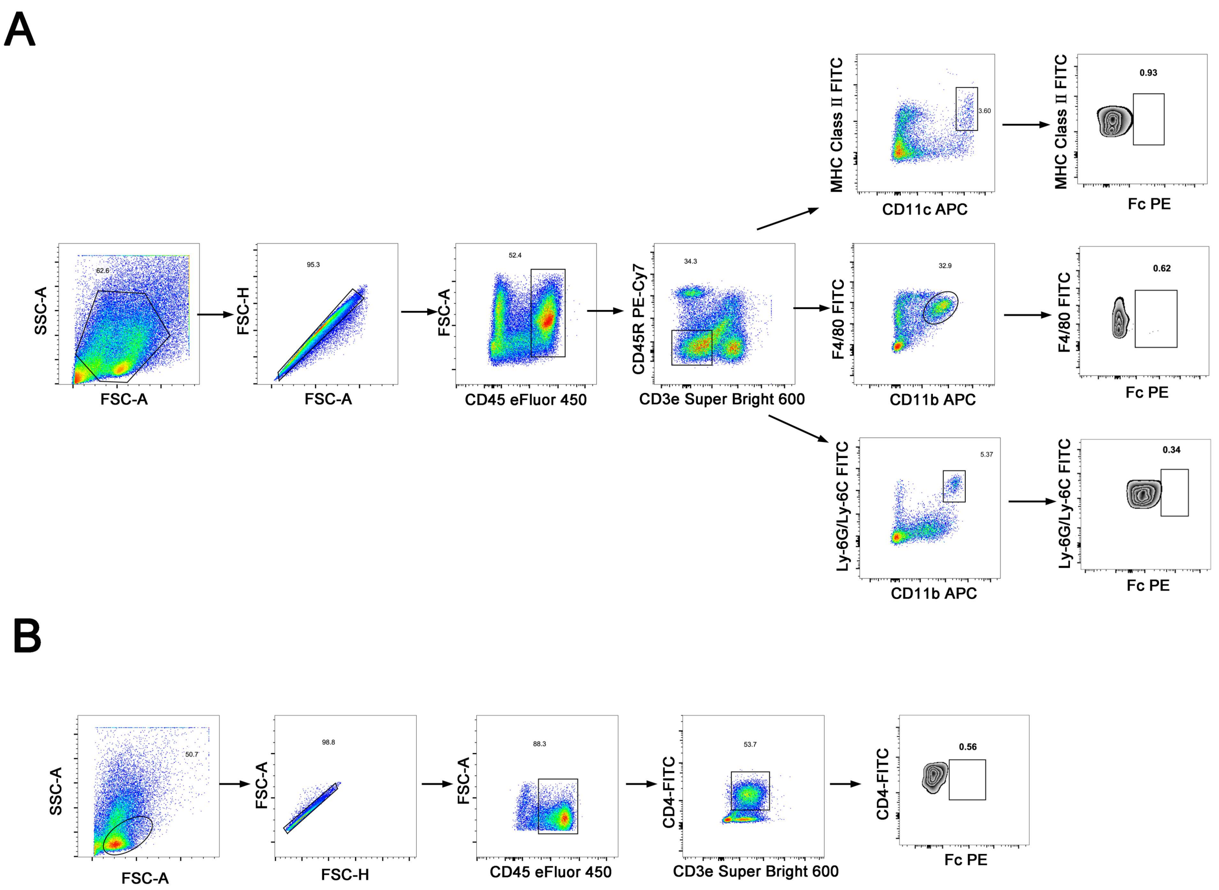

Supplementary Fig. 21 Gating strategy for the FACS tests. Panel A was applied for Supplementary Fig. 10B-10D and Supplementary Fig. 11B-11D. Panel B was applied for Supplementary Fig. 10A and Supplementary Fig. 11A.

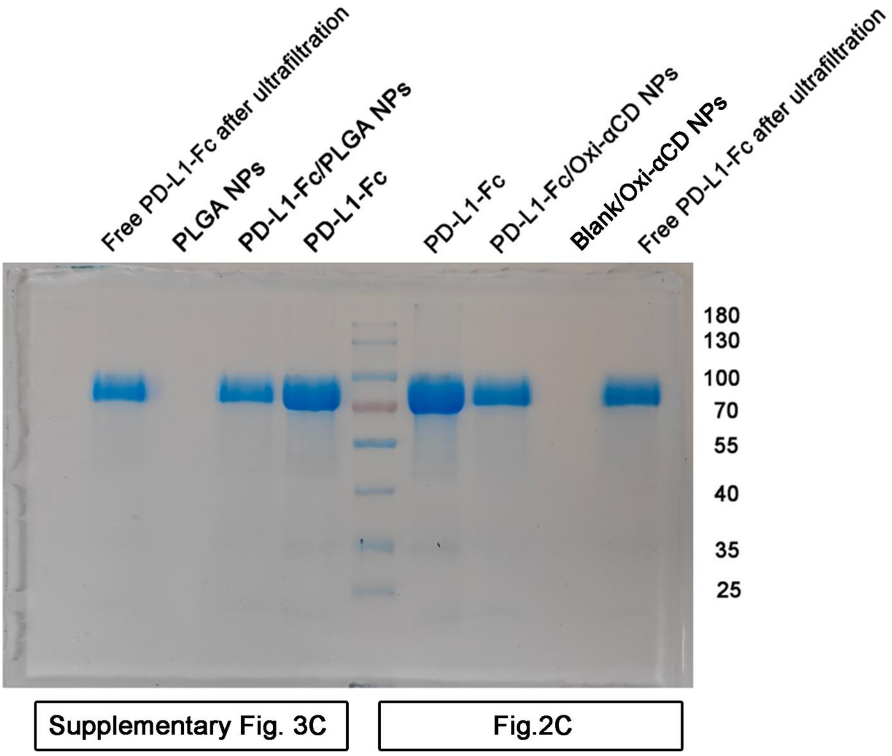

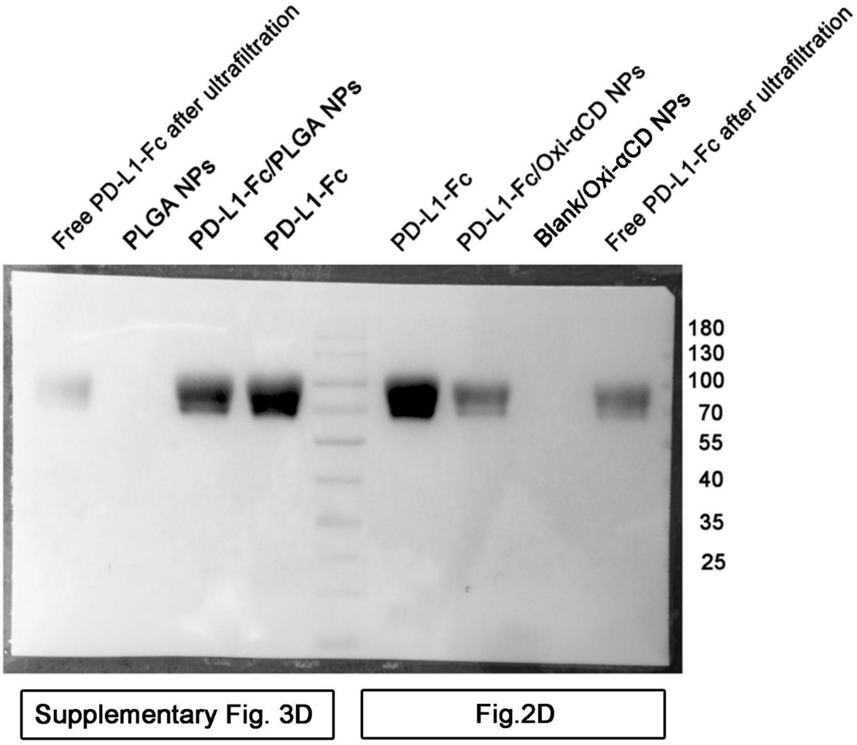

Supplementary Fig. 22 Uncropped scans of gels and western blot with marked molecular weight distributions for related figures. NPs nanoparticles.

Supplementary Table 1. Physicochemical characterization of nanoparticles

Nanoformulation	Hydrodynamic size (nm)	Zeta potential (mV)	PDI	PD-L1-Fc loading (%)
Blank/Oxi-αCD	150.6±3.2	-20.8±0.5	0.115±0.062	-
PD-L1-Fc/Oxi-αCD	221.9±2.7	-18.2±1.4	0.200±0.013	45.33%±3.38
Blank/PLGA	118.5±2.5	-26.8±1.7	0.101±0.023	-
PD-L1-Fc/PLGA	178.6±3.4	-22.6±0.5	0.158±0.011	58.17%±5.19

Supplementary Methods

Antibodies

The PD-L1/CD274 polyclonal antibody [BC074984] for western blotting (17952-1-AP, 1:1000) was purchased from Proteintech Group, Inc. (Wuhan, China). Super Bright 600-conjugated anti-mouse CD3e antibody [145-2C11] (63-0031-82, 1:100), eFluor 450-conjugated anti-mouse CD45 antibody [30-F11] (48-0451-82, 1:200), PE-Cyanine7-conjugated anti-mouse CD45R antibody [RA3-6B2] (25-0452-81, 1:200), PE-conjugated anti-mouse F4/80 antibody [BM8] (12-4801-82, 1:100), PE-conjugated anti-human IgG Fc antibody (12-4998-82, 1:100), FITC-conjugated anti-human IgG Fc antibody (A18818, 1:1000), FITC-conjugated anti-mouse CD4 antibody [GK1.5] (11-0041-82, 1:200), FITC-conjugated anti-mouse CD11b antibody [M1/70] (11-0112-81, 1:400), FITC-conjugated anti-mouse CD11c antibody [N418] (11-0114-81, 1:200), FITC-conjugated anti-mouse Ly-6G/Ly-6C antibody [RB6-8C5] (11-5931-81, 1:100), FITC-conjugated anti-mouse MHC Class II antibody [M5/114.15.2] (11-5321-82, 1:200), FITC-conjugated anti-mouse F4/80 antibody [BM8] (11-4801-82, 1:100), PE-conjugated anti-mouse TCRβ antibody [H57-597](12-5961-82, 1:200), APC-conjugated anti-mouse FOXP3 antibody [FJK-16s] (17-5773-80, 1:20), APC-conjugated anti-mouse CXCR3 antibody [CXCR3-173] (17-1831-82, 1:40), APC-conjugated anti-mouse CXCR5 antibody [SPRCL5] (17-7185-80, 1:40), APC-conjugated anti-mouse CD11b antibody [M1/70] (17-0112-81, 1:200), APC-conjugated anti-mouse CD11c antibody [N418] (17-0114-81, 1:200), and APC-conjugated anti-mouse MHC Class II antibody [M5/114.15.2] (17-5321-82, 1:200) for flow cytometry were purchased from Thermo Fisher Scientific Inc. (Waltham, MA, USA). Anti-mouse CD3ε antibody [145-2C11] (100340, 1:2500) was purchased from BioLegend Co., Ltd. (San Diego, California, USA).

Detect of PD-L1-Fc on nanoparticles

PD-L1-Fc-loaded nanoparticles and their blank control nanoparticles were diluted in SDS loading buffer in the presence of 50 mM DTT and loaded on a 10% polyacrylamide gel for electrophoresis. The samples separated by the gel were stained with Coomassie blue (Bio-Rad, USA) or transferred to a PVDF membrane for further western blot analysis. The membrane was blocked with 5% skim milk in TBS with 0.1% Tween-20 for 2 hours at RT and incubated with PD-L1/CD274 polyclonal antibody (1:1000) overnight at 4 °C.

In vitro hydrolysis and release study

In vitro hydrolysis of PD-L1-Fc-loaded nanoparticles or their blank control nanoparticles was performed at 37 °C in ultrapure water with or without 1.0 mM hydrogen peroxide (H_2O_2). The hydrolysis curve of the nanoparticles was calculated based on the transmittance values at 490 nm at various time points (0, 10, 20, 30, 60, 120, 240 and 360 min).

To detect the in vitro release of PD-L1-Fc, 1 mg of freshly fabricated Cy5-PD-L1-Fc-loaded nanoparticles was immersed in 1 mL of ultrapure water with or without different concentrations of H_2O_2. At various time points, the released Cy5-PD-L1-Fc in H_2O_2 was removed by a 100-kDa ultrafiltration spin column and then measured by fluorescence spectroscopy to quantify the concentration of PD-L1-Fc in the release medium. Twenty microliters of released Cy5-PD-L1-Fc in different concentrations of H_2O_2 (0 μM, 100 μM, 200 μM or 500 μM H_2O_2) was incubated with anti-CD3-activated spleen lymphocytes for 8 h. After washing 3 times with PBS and staining with DAPI for 15 min, the cells were visualized with CLSM to detect Cy5 fluorescence.

Detection of H_2O_2 level

To evaluate the H_2O_2-scavenging capacity of the nanoparticles, 1 mg of PD-L1-Fc/Oxi-αCD nanoparticles or PD-L1-Fc/PLGA nanoparticles was incubated with 1 mM H_2O_2 for 360 min. A hydrogen peroxide assay kit (S0038, Beyotime) was used to determine the concentration of residual H_2O_2. The H_2O_2 scavenging ability of the nanoparticles was calculated based on the transmittance values at 560 nm at various time points (0, 10, 20, 30, 60, 120, 240 and 360 min).

The H_2O_2 level in the colonic tissue of healthy or colitis mice treated with different formulations were measured following the protocols provided by the hydrogen peroxide assay kit. Briefly, colon tissues were homogenized in PBS buffer (pH 7.4) and centrifuged at 12,000 × g for 10 min at 4 °C to collect the supernatant. The total protein concentration in the supernatant was measured by the BCA method. The H_2O_2 level are expressed as mmol per mg protein.

Serum stability of the nanoparticles

To examine the serum stability of the nanoparticles, PD-L1-Fc-loaded nanoparticles were incubated in ultrapure water with 10% FBS at 37 °C, and the size and PDI of the PD-L1-Fc/Oxi-αCD nanoparticles and PD-L1-Fc/PLGA nanoparticles were monitored at 0, 2, 4, 6, 8, 12, and 24 h by Malvern Zetasizer Nano ZSP equipment (Malvern, U.K.). The nanoparticles incubated with 10% FBS for 0 h and 24 h were used to treat anti-CD3-activated lymphocytes for 8 h, and the protein stability was tested by assessing the binding to lymphocytes using flow cytometry. Human IgG1 Fc was set as the negative control to exclude the Fc fragment binding to activated lymphocytes.

In vitro binding of PD-L1-Fc

Mouse spleen lymphocytes were stimulated with 1 μg/mL anti-mouse CD3ε antibody (1:2500), which had been precoated on a 96-well plate. Human IgG1 Fc (2.5 μg/mL), PD-L1-Fc (2.5 μg/mL), or nanoparticles loaded with 2.5 μg/mL PD-L1-Fc were added to activated mouse spleen lymphocytes. After treatment, the cells were washed with PBS and stained with PE-conjugated human IgG Fc antibody (1:100). The binding of PD-L1-Fc with lymphocytes in different treatment groups was assessed by analyzing the fluorescence of

the cells by an ACEA NovoCyte flow cytometer (Agilent, USA) and NovoExpress software (Agilent, USA).

CCK-8 assays

Mouse spleen lymphocytes were stimulated with 1 μg/mL anti-mouse CD3ε antibody (1:2500), which had been precoated on a 96-well plate. Human IgG1 Fc (2.5 μg/mL), PD-L1-Fc (2.5 μg/mL), or nanoparticles loaded with 2.5 μg/mL PD-L1-Fc were added to activated mouse spleen lymphocytes. Then, 10 μL/well of CCK-8 solution was added to the cells at various time points (0, 12, 24, 48 and 64 hours) and incubated for 4 hours at 37 °C. The absorbance of cells per well was measured at 460 nm using a microplate reader (Bio-Rad, USA).
