# Supplementary material for: Targeted delivery of Fc-fused PD-L1 for effective management of acute and chronic colitis
Source: Nat Commun. 2024 Feb 23;15:1673. doi: 10.1038/s41467-024-46025-0 (PMC10891058; doi:10.1038/s41467-024-46025-0)
Supplement: Supplementary file 3 — Reporting Summary [file 41467_2024_46025_MOESM3_ESM.pdf]

nature portfolio

Corresponding author(s): Lei Chen, Dinglin Zhang

Last updated by author(s): Feb 2, 2024

Reporting Summary

Nature Portfolio wishes to improve the reproducibility of the work that we publish. This form provides structure for consistency and transparency in reporting. For further information on Nature Portfolio policies, see our Editorial Policies and the Editorial Policy Checklist.

Statistics

For all statistical analyses, confirm that the following items are present in the figure legend, table legend, main text, or Methods section.

n/a	Confirmed	
☐	☒	The exact sample size (*n*) for each experimental group/condition, given as a discrete number and unit of measurement
☐	☒	A statement on whether measurements were taken from distinct samples or whether the same sample was measured repeatedly
☐	☒	The statistical test(s) used AND whether they are one- or two-sided<br>*Only common tests should be described solely by name; describe more complex techniques in the Methods section.*
☒	☐	A description of all covariates tested
☐	☒	A description of any assumptions or corrections, such as tests of normality and adjustment for multiple comparisons
☐	☒	A full description of the statistical parameters including central tendency (e.g. means) or other basic estimates (e.g. regression coefficient) AND variation (e.g. standard deviation) or associated estimates of uncertainty (e.g. confidence intervals)
☒	☐	For null hypothesis testing, the test statistic (e.g. *F*, *t*, *r*) with confidence intervals, effect sizes, degrees of freedom and *P* value noted<br>*Give P values as exact values whenever suitable.*
☒	☐	For Bayesian analysis, information on the choice of priors and Markov chain Monte Carlo settings
☒	☐	For hierarchical and complex designs, identification of the appropriate level for tests and full reporting of outcomes
☒	☐	Estimates of effect sizes (e.g. Cohen's *d*, Pearson's *r*), indicating how they were calculated

Our web collection on statistics for biologists contains articles on many of the points above.

Software and code

Policy information about availability of computer code

Data collection	TECNAI-10 microscope (Philips, Netherlands), FIB-SEM microscope (Crossbeam 340, Zeiss), Malvern Zetasizer Nano ZSP, Philips TECNAI-10 microscope, Image Lab 3.0, Aniview 100 Living Image systerm, BD LSRFortessaTM flow cytometer, Agilent ACEA NovoCyte flow cytometer, Zeiss LSM880 laser scanning confocal microscope, Hitachi fluorescence spectroscopy F-7000, Bio-Rad microplate reader, Thermo Trace 1300 gas chromatograph (Waltham, MA, USA)
Data analysis	statistical calculations were performed using Graphpad prism 8.0 (San Diego, CA, USA), Flow cytometry results were analyzed by Agilent NovoExpress software (Agilent, USA) or FlowJo V10 (Treestar, Ashland, OR, USA), Fluorescence images were analyzed by Image J 1.53 (National Institutes of Health, USA). 16S rRNA sequence data were analyzed via QIIME2 (2021.11) (https://qiime2.org/) and DADA2 R package (version 1.30.0).

For manuscripts utilizing custom algorithms or software that are central to the research but not yet described in published literature, software must be made available to editors and reviewers. We strongly encourage code deposition in a community repository (e.g. GitHub). See the Nature Portfolio guidelines for submitting code & software for further information.

Data

Policy information about availability of data

All manuscripts must include a data availability statement. This statement should provide the following information, where applicable:

- Accession codes, unique identifiers, or web links for publicly available datasets
- A description of any restrictions on data availability
- For clinical datasets or third party data, please ensure that the statement adheres to our policy

The raw 16S rRNA gene sequences data generated in this study have been deposited in the Genome Sequence Archive (GSA) database60, under accession code CRA014490 (https://ngdc.cncb.ac.cn/gsa/browse). The authors declare that all data provided in this study are available within the Article, Supplementary Information or Source data file. Source data are provided with this paper.

Research involving human participants, their data, or biological material

Policy information about studies with human participants or human data. See also policy information about sex, gender (identity/presentation), and sexual orientation and race, ethnicity and racism.

Reporting on sex and gender	N/A
Reporting on race, ethnicity, or other socially relevant groupings	N/A
Population characteristics	N/A
Recruitment	N/A
Ethics oversight	N/A

Note that full information on the approval of the study protocol must also be provided in the manuscript.

Field-specific reporting

Please select the one below that is the best fit for your research. If you are not sure, read the appropriate sections before making your selection.

☒ Life sciences ☐ Behavioural & social sciences ☐ Ecological, evolutionary & environmental sciences

For a reference copy of the document with all sections, see nature.com/documents/nr-reporting-summary-flat.pdf

Life sciences study design

All studies must disclose on these points even when the disclosure is negative.

Sample size	Sample size was chosen to ensure reproducibility of the experiments in accordance with the replacement, reduction and refinement principles of animal ethics regulation. Sample sizes employed in the study were refered to several studies related to mouse model of colitis (J Neuroinflammation. 2021,18(1):153; Adv Sci. 2019, 6(18):1900610).
Data exclusions	No data was excluded in this study.
Replication	All the data were reliably reproduced. All experiments contained at least three independent replicates. Three independent samples were performed to cellular level experiments.
Randomization	All samples were randomly allocated into experimental groups.
Blinding	No specific double-blinding was used throughout experiments, because most of experiments need multiple treatments (including mouse modeling, formulation, tissue collection, cell isolated, and so on) and the participation of multiple investigators. In animal experiments and cell experiments, investigators were blinded to group allocation during data collection and/or analysis. The investigators participated in data collection and/or analysis are different from investigators participated in experiments.

Reporting for specific materials, systems and methods

We require information from authors about some types of materials, experimental systems and methods used in many studies. Here, indicate whether each material, system or method listed is relevant to your study. If you are not sure if a list item applies to your research, read the appropriate section before selecting a response.

Materials & experimental systems

n/a	Involved in the study	
☐	☒	Antibodies
☒	☐	Eukaryotic cell lines
☒	☐	Palaeontology and archaeology
☐	☒	Animals and other organisms
☒	☐	Clinical data
☒	☐	Dual use research of concern
☒	☐	Plants

Methods

n/a	Involved in the study	
☒	☐	ChIP-seq
☐	☒	Flow cytometry
☒	☐	MRI-based neuroimaging

Antibodies

Antibodies used

The PD-L1/CD274 polyclonal antibody [BC074984] for western blotting (17952-1-AP, 1:1000) was purchased from Proteintech Group, Inc. (Wuhan, China). Super Bright 600-conjugated anti-mouse CD3e antibody [145-2C11] (63-0031-82, 1:100), eFluor 450-conjugated anti-mouse CD45 antibody [30-F11] (48-0451-82, 1:200), PE-Cyanine7-conjugated anti-mouse CD45R antibody [RA3-6B2] (25-0452-81, 1:200), PE-conjugated anti-mouse F4/80 antibody [BM8] (12-4801-82, 1:100), PE-conjugated anti-human IgG Fc antibody (12-4998-82, 1:100), FITC-conjugated anti-human IgG Fc antibody (A18818, 1:1000), FITC-conjugated anti-mouse CD4 antibody [GK1.5] (11-0041-82, 1:200), FITC-conjugated anti-mouse CD11b antibody [M1/70] (11-0112-81, 1:400), FITC-conjugated anti-mouse CD11c antibody [N418] (11-0114-81, 1:200), FITC-conjugated anti-mouse Ly-6G/Ly-6C antibody [RB6-8C5] (11-5931-81, 1:100), FITC-conjugated anti-mouse MHC Class II antibody [M5/114.15.2] (11-5321-82, 1:200), FITC-conjugated anti-mouse F4/80 antibody [BM8] (11-4801-82, 1:100), PE-conjugated anti-mouse TCRβ antibody [H57-597](12-5961-82, 1:200), APC-conjugated anti-mouse Foxp3 antibody [FJK-16s] (17-5773-80, 1:20), APC-conjugated anti-mouse CXCR3 antibody [CXCR3-173] (17-1831-82, 1:40), APC-conjugated anti-mouse CXCR5 antibody [SPRCL5] (17-7185-80, 1:40), APC-conjugated anti-mouse CD11b antibody [M1/70] (17-0112-81, 1:200), APC-conjugated anti-mouse CD11c antibody [N418] (17-0114-81, 1:200), and APC-conjugated anti-mouse MHC Class II antibody [M5/114.15.2] (17-5321-82, 1:200) for flow cytometry were purchased from Thermo Fisher Scientific Inc. (Waltham, MA, USA). Anti-mouse CD3ε antibody [145-2C11] (100340, 1:2500) was purchased from BioLegend Co., Ltd. (San Diego, California, USA).

Validation

All primary antibodies were commercially available and were validated by the supplier. All antibodies were used in the study according to theprofile of manufacturers. All validation statements are available on the antibody websites, respectively.
1. PD-L1/CD274 polyclonal antibody [BC074984] (17952-1-AP, Proteintech)/Manufacturer detected this antibody in human placenta tissue and A549 cells for western, in human tonsillitis tissue for immunohistochemistry, in HEK-293 cells for Immunofluorescent analysis. https://www.ptgcn.com/products/CD274-Antibody-17952-1-AP.htm.
2. Super Bright 600-conjugated anti-mouse CD3e antibody [145-2C11] (63-0031-82, Thermo Fisher)/Manufacturer detected this antibody in mouse splenocytes for Flow. https://www.thermofisher.cn/cn/en/antibody/product/CD3e-Antibody-clone-145-2C11-Monoclonal/63-0031-82
3. eFluor 450-conjugated anti-mouse CD45 antibody [30-F11] (48-0451-82, Thermo Fisher)/Manufacturer detected this antibody in C57BL/6 bone marrow cells for Flow. https://www.thermofisher.cn/cn/en/antibody/product/CD45-Antibody-clone-30-F11-Monoclonal/48-0451-82
4. PE-Cyanine7-conjugated anti-mouse CD45R antibody [RA3-6B2] (25-0452-81, Thermo Fisher)/Manufacturer detected this antibody in mouse splenocytes and bone marrow cells for Flow. https://www.thermofisher.cn/cn/en/antibody/product/CD45R-B220-Antibody-clone-RA3-6B2-Monoclonal/25-0452-81
5. PE-conjugated anti-mouse F4/80 antibody [BM8] (12-4801-82, Thermo Fisher)/Manufacturer detected this antibody in C57Bl/6 resident peritoneal exudate cells for Flow. https://www.thermofisher.cn/cn/en/antibody/product/F4-80-Antibody-clone-BM8-Monoclonal/12-4801-82
6. FITC-conjugated anti-mouse CD4 antibody [GK1.5] (11-0041-82, Thermo Fisher)/Manufacturer detected this antibody in mouse splenocytes and mouse thymocytes for Flow. https://www.thermofisher.cn/cn/en/antibody/product/CD4-Antibody-clone-GK1-5-Monoclonal/11-0041-82
7. FITC-conjugated anti-mouse CD11b antibody [M1/70] (11-0112-81, Thermo Fisher)/Manufacturer detected this antibody in mouse bone marrow cells for Flow. https://www.thermofisher.cn/cn/en/antibody/product/CD11b-Antibody-clone-M1-70-Monoclonal/11-0112-81
8. FITC-conjugated anti-mouse CD11c antibody [N418] (11-0114-81, Thermo Fisher)/Manufacturer detected this antibody in C57BL/6 splenocytes for Flow. https://www.thermofisher.cn/cn/en/antibody/product/CD11c-Antibody-clone-N418-Monoclonal/11-0114-81
9. FITC-conjugated anti-mouse Ly-6G/Ly-6C antibody [RB6-8C5] (11-5931-81, Thermo Fisher)/Manufacturer detected this antibody in mouse bone marrow cells for Flow. https://www.thermofisher.cn/cn/en/antibody/product/Ly-6G-Ly-6C-Antibody-clone-RB6-8C5-Monoclonal/11-5931-81
10. FITC-conjugated anti-mouse MHC Class II antibody [M5/114.15.2] (11-5321-82, Thermo Fisher)/Manufacturer detected this antibody in C57BL/6 splenocytes for Flow. https://www.thermofisher.cn/cn/en/antibody/product/MHC-Class-II-I-A-I-E-Antibody-clone-M5-114-15-2-Monoclonal/11-5321-82
11. FITC-conjugated anti-mouse F4/80 antibody [BM8] (11-4801-82, Thermo Fisher)/Manufacturer detected this antibody in mouse resident peritoneal exudate cells for Flow. https://www.thermofisher.cn/cn/en/antibody/product/F4-80-Antibody-clone-BM8-Monoclonal/11-4801-82
12. PE-conjugated anti-mouse TCRβ antibody [H57-597](12-5961-82, Thermo Fisher)/Manufacturer detected this antibody in BALB/c splenocytes for Flow. https://www.thermofisher.cn/cn/en/antibody/product/TCR-beta-Antibody-clone-H57-597-Monoclonal/12-5961-82
13. APC-conjugated anti-mouse Foxp3 antibody [FJK-16s] (17-5773-80, Thermo Fisher)/Manufacturer detected this antibody in mouse splenocytes for Flow. https://www.thermofisher.cn/cn/en/antibody/product/FOXP3-Antibody-clone-FJK-16s-Monoclonal/17-5773-80
14. APC-conjugated anti-mouse CXCR3 antibody [CXCR3-173] (17-1831-82, Thermo Fisher)/Manufacturer detected this antibody in

C57Bl/6 splenocytes for Flow. https://www.thermofisher.cn/cn/en/antibody/product/CD183-CXCR3-Antibody-clone-CXCR3-173-Monoclonal/17-1831-82
15. APC-conjugated anti-mouse CXCR5 antibody [SPRCL5] (17-7185-80, Thermo Fisher)/Manufacturer detected this antibody in C57Bl/6 splenocytes for Flow. https://www.thermofisher.cn/cn/en/antibody/product/CD185-CXCR5-Antibody-clone-SPRCL5-Monoclonal/17-7185-80
16. APC-conjugated anti-mouse CD11b antibody [M1/70] (17-0112-81, Thermo Fisher)/Manufacturer detected this antibody in mouse bone marrow cells for Flow. https://www.thermofisher.cn/cn/en/antibody/product/CD11b-Antibody-clone-M1-70-Monoclonal/17-0112-81
17. APC-conjugated anti-mouse CD11c antibody [N418] (17-0114-81, Thermo Fisher)/Manufacturer detected this antibody in C57BL/6 splenocytes for Flow. https://www.thermofisher.cn/cn/zh/antibody/product/CD11c-Antibody-clone-N418-Monoclonal/17-0114-81
18. APC-conjugated anti-mouse MHC Class Ⅱ antibody [M5/114.15.2] (17-5321-82, Thermo Fisher)/Manufacturer detected this antibody in C57BL/6 splenocytes for Flow. https://www.thermofisher.cn/cn/en/antibody/product/MHC-Class-II-I-A-I-E-Antibody-clone-M5-114-15-2-Monoclonal/17-5321-82
19. Anti-mouse CD3ε antibody [145-2C11] (100340, BioLegend)/ Manufacturer detected this antibody in C57BL/6 splenocytes for Flow.https://www.biolegend.com/en-gb/products/ultra-leaf-purified-anti-mouse-cd3epsilon-antibody-7722?GroupID=BLG6744

Animals and other research organisms

Policy information about studies involving animals; ARRIVE guidelines recommended for reporting animal research, and Sex and Gender in Research

Laboratory animals	C57BL/6J(female, 6 ~8 weeks, 18~20 g, stock number: N000013) uesed in colitis models were bred and housed under SPF conditions and maintained in microisolator cages on individually ventilated cage-racks filled with aspen chip bedding at a room temperature of 20–22 °C in a humidity-controlled (45–65 °C) environment under a 12-h light/dark cycle, with ad libitum access to autoclaved rodent chow and autoclaved water. All diets of mice were provided by Laboratory Animal Science of Army Medical University. All mice were acclimatized for one week before further experiments. the female C57BL/6J mice were used to all experiments. The experimental mice and control mice were bred separately in different cages.
Wild animals	No wild animals were used in this study.
Reporting on sex	We did not consider the influence of sex in the study design. Female mice were used for all the animal assay as reported in the literature studies.
Field-collected samples	No field-collected samples were used in this study.
Ethics oversight	Study protocols were approved by the Laboratory Animal Welfare and Ethics Committee of the Army Medical University (Chongqing, China). Approval number: AMUWEC20212167

Note that full information on the approval of the study protocol must also be provided in the manuscript.

Plants

Seed stocks	*Report on the source of all seed stocks or other plant material used. If applicable, state the seed stock centre and catalogue number. If plant specimens were collected from the field, describe the collection location, date and sampling procedures.*
Novel plant genotypes	*Describe the methods by which all novel plant genotypes were produced. This includes those generated by transgenic approaches, gene editing, chemical/radiation-based mutagenesis and hybridization. For transgenic lines, describe the transformation method, the number of independent lines analyzed and the generation upon which experiments were performed. For gene-edited lines, describe the editor used, the endogenous sequence targeted for editing, the targeting guide RNA sequence (if applicable) and how the editor was applied.*
Authentication	*Describe any authentication procedures for each seed stock used or novel genotype generated. Describe any experiments used to assess the effect of a mutation and, where applicable, how potential secondary effects (e.g. second site T-DNA insertions, mosiacism, off-target gene editing) were examined.*

Flow Cytometry

Plots

Confirm that:

☒ The axis labels state the marker and fluorochrome used (e.g. CD4-FITC).

☒ The axis scales are clearly visible. Include numbers along axes only for bottom left plot of group (a 'group' is an analysis of identical markers).

☒ All plots are contour plots with outliers or pseudocolor plots.

☒ A numerical value for number of cells or percentage (with statistics) is provided.

Methodology

Sample preparation	Mouse spleen lymphocytes were isolated by using the mouse spleen lymphocyte cell isolation kit (Solarbio, China) and then

Sample preparation	stimulated with 1 μg/mL anti-mouse CD3ε antibody, which had been precoated on a 96-well plate. Human IgG1 Fc (2.5 μg/mL), PD-L1-Fc (2.5 μg/mL), or nanoparticles loaded with 2.5 μg/mL PD-L1-Fc were added to activated mouse spleen lymphocytes. After treatment, the cells were washed with PBS and stained with PE-conjugated human IgG Fc antibody. The binding of PD-L1-Fc or PD-L1-Fc-loaded NPs to lymphocytes was assessed by analyzing the fluorescence of the cells by ACEA NovoCyte flow cytometer (Agilent, USA) and NovoExpress software (Agilent, USA).To isolate lymphocytes from colonic lamina propria, the colons were immediately isolated and placed in precooled PBS. After removing adipose and mesenteric tissues, the intestine was dissected longitudinally along one side of the mesentery, rinsed, and cut into segments of approximately 0.5-1.0 cm laterally. The intestinal segments were placed in a washing solution consisting of 1 mM DTT, 5 mM EDTA and 10 mM HEPES; shaken at 250 rpm for 30 min at 37 ℃; and filtered through a 100-μm cell strainer. A digestion solution containing 150 μg/mL DNase I and 200 U/ml collagenase IV was added to the filtered cell suspension and shaken at 250 rpm for 60 min at 37 °C. Then, the cells were filtered through a 100-μm cell strainer and centrifuged at 400 × g for 5 min at room temperature. The cell precipitate was resuspended in 1 mL of 40% Percoll solution, and the lymphocytes were isolated via a Percoll density gradient. The isolated lymphocytes were then resuspended with PBS and stained with antibodies for 30 min at 4 ℃. Flow cytometric analysis was performed on a BD LSRFortessaTM flow cytometer (BD, USA). The data were analyzed using FlowJo v10 software.
Instrument	ACEA NovoCyte flow cytometer and BD LSRFortessaTM flow cytometer for data collection.
Software	NovoExpress software and FlowJo v10 software for data analysis.
Cell population abundance	At least 10000 cells were used for flow cytometric analysis.
Gating strategy	Cells were gated on FSC/SSC in general.

☒ Tick this box to confirm that a figure exemplifying the gating strategy is provided in the Supplementary Information.
